# Supplementary figures and images for: Molecular and anatomical characterization of parabrachial neurons and their axonal projections
Source: eLife. 2022 Nov 1;11:e81868. doi: 10.7554/eLife.81868 (PMC9668336; doi:10.7554/eLife.81868)

cluster-specific features

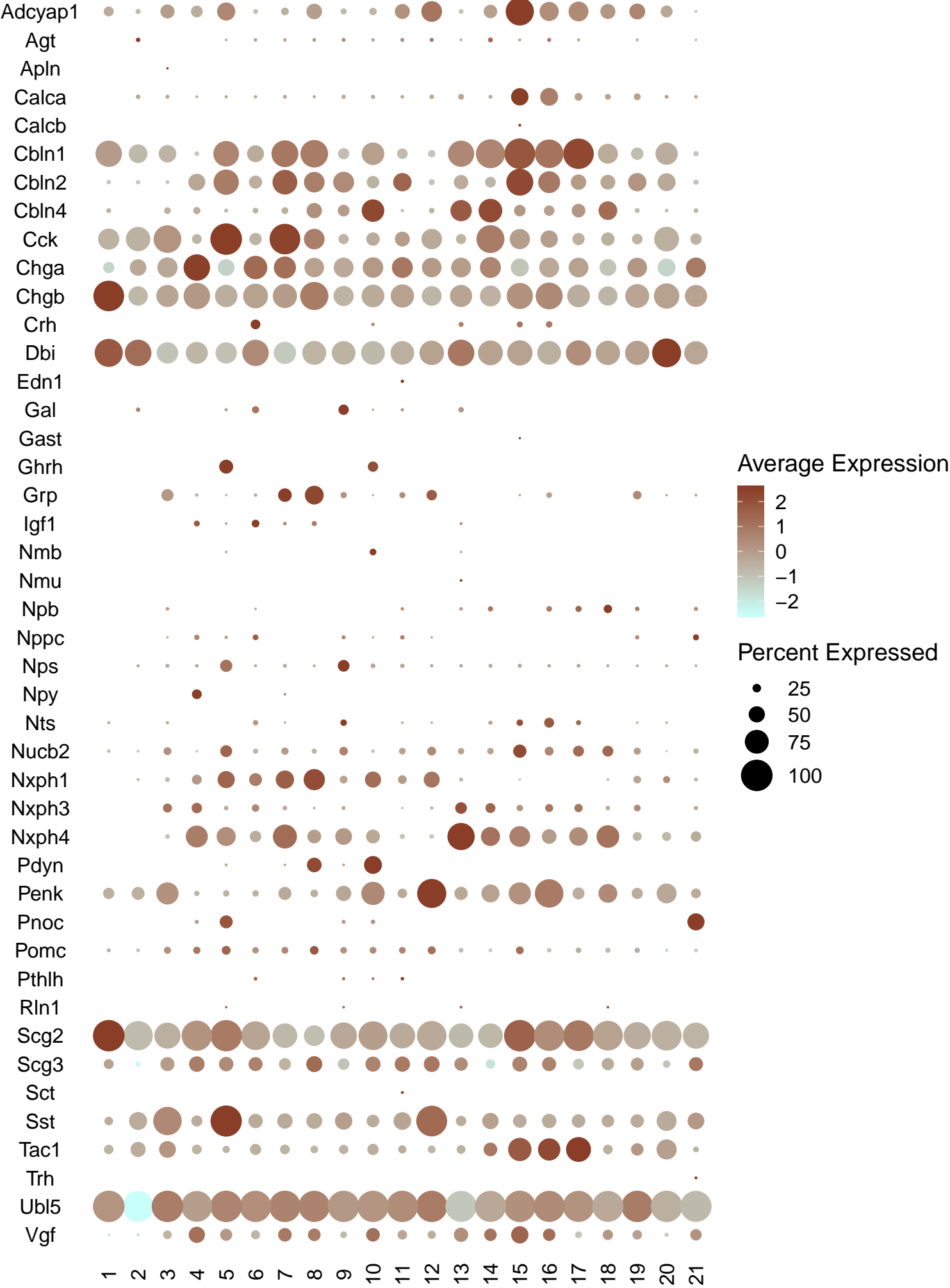

Supplement: Supplementary file 3. [file elife-81868-supp3.pdf]

cluster-specific features

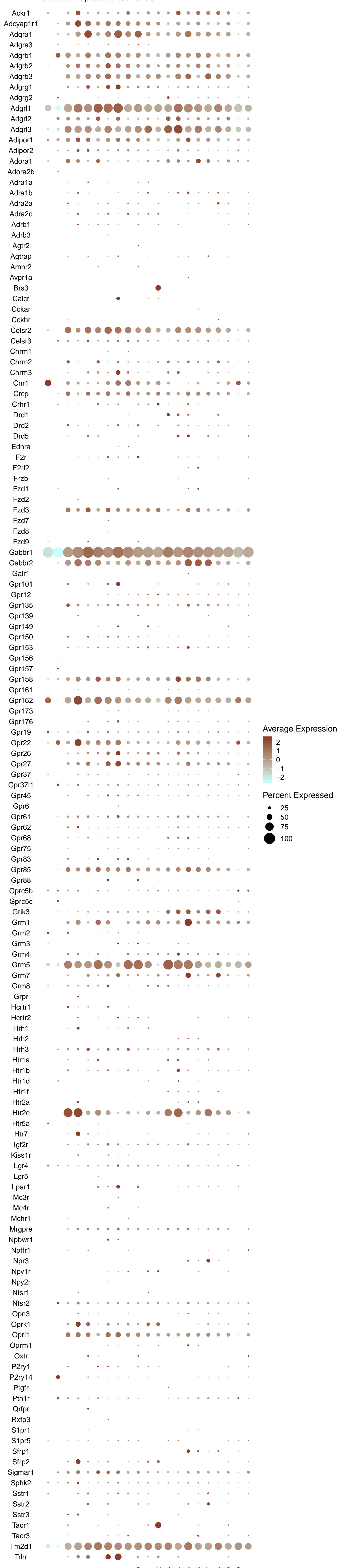

Supplement: Supplementary file 4. [file elife-81868-supp4.pdf]
