# Supplementary material for: Molecular and anatomical characterization of parabrachial neurons and their axonal projections
Source: eLife. 2022 Nov 1;11:e81868. doi: 10.7554/eLife.81868 (PMC9668336; doi:10.7554/eLife.81868)
Supplement: Supplementary file 5. [file elife-81868-supp5.pptx]

## Slide 1
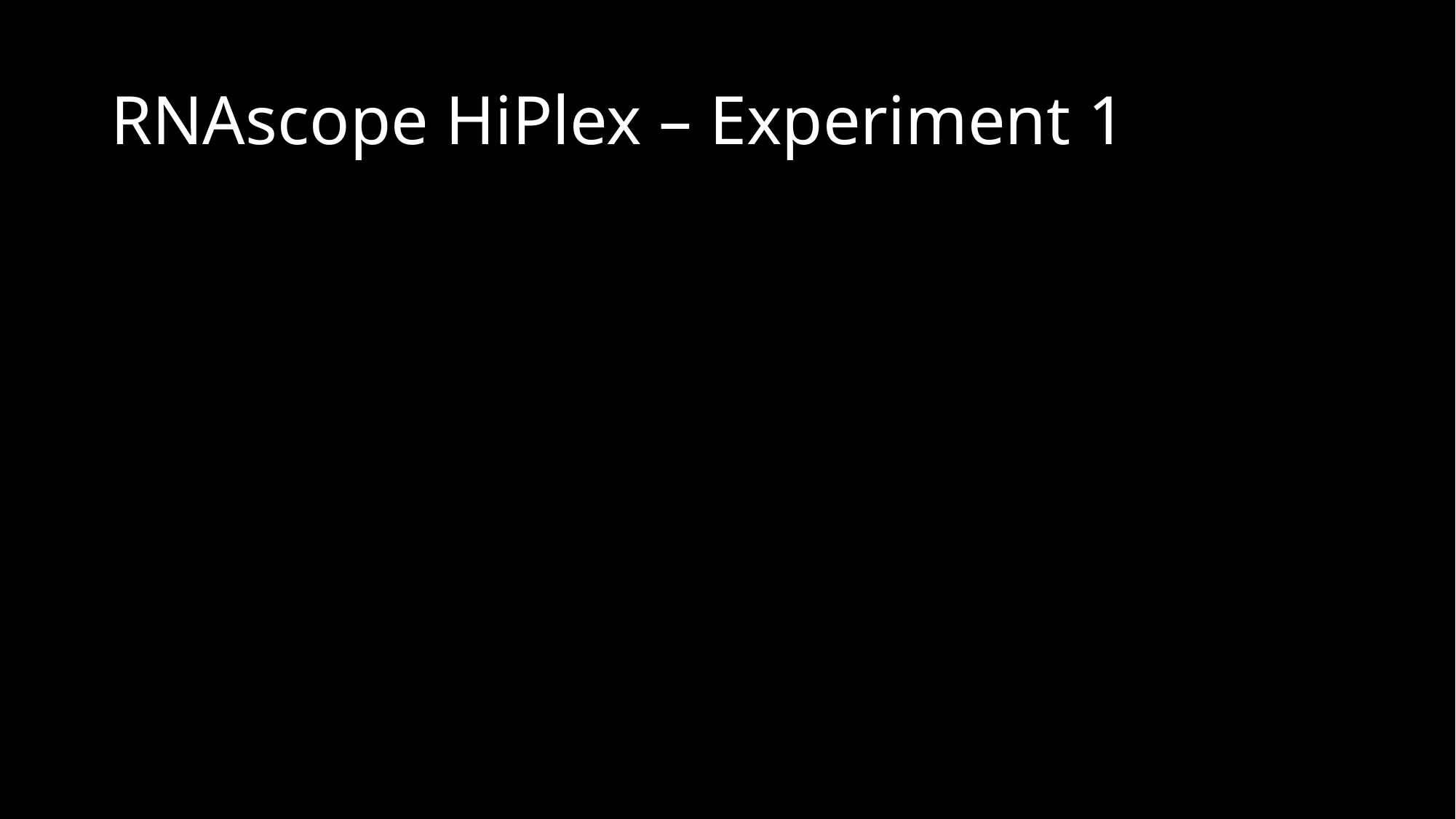

# RNAscope HiPlex – Experiment 1

## Slide 2
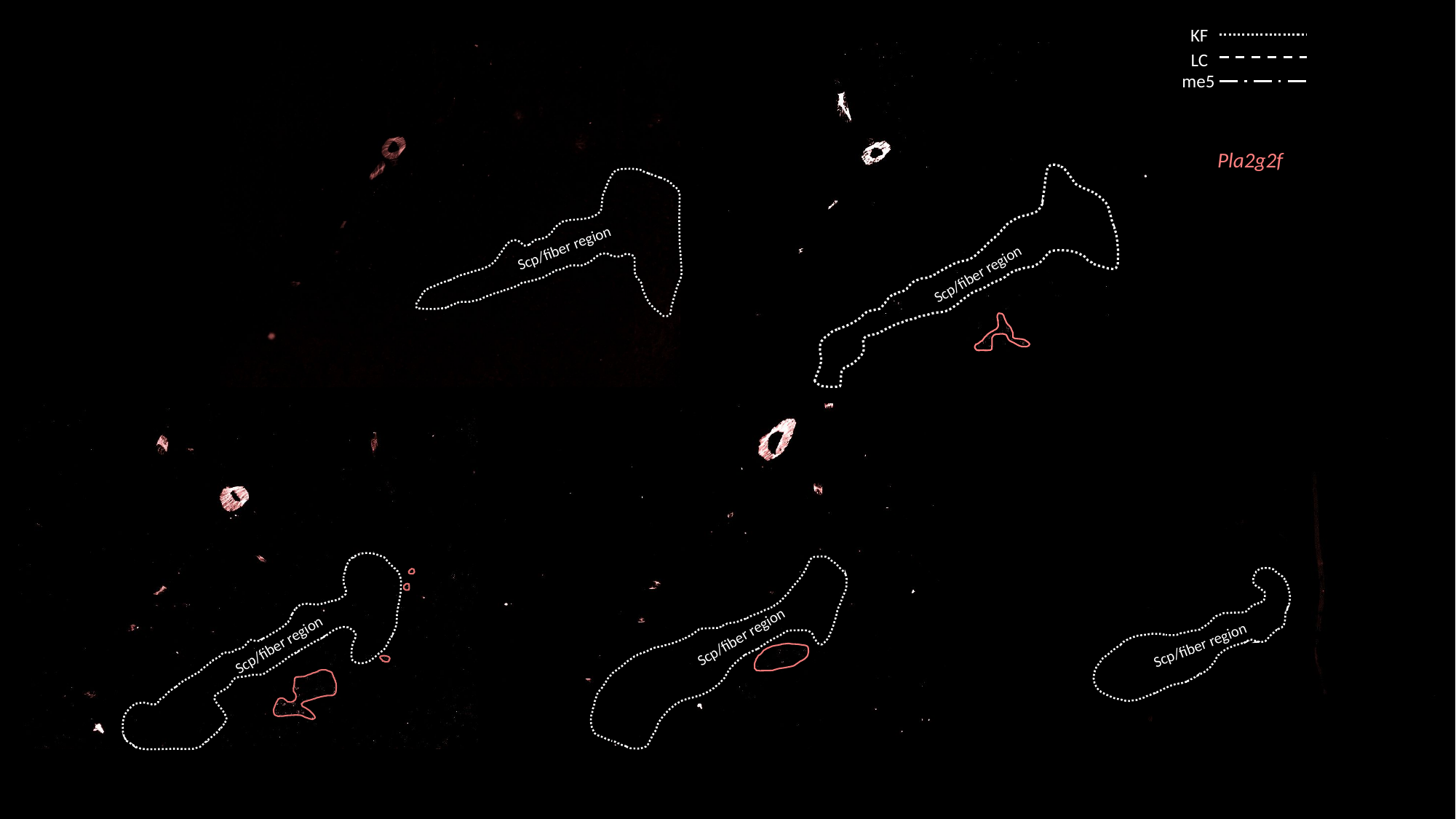

KF
LC
me5
Pla2g2f
Scp/fiber region
Scp/fiber region
Scp/fiber region
Scp/fiber region
Scp/fiber region

## Slide 3
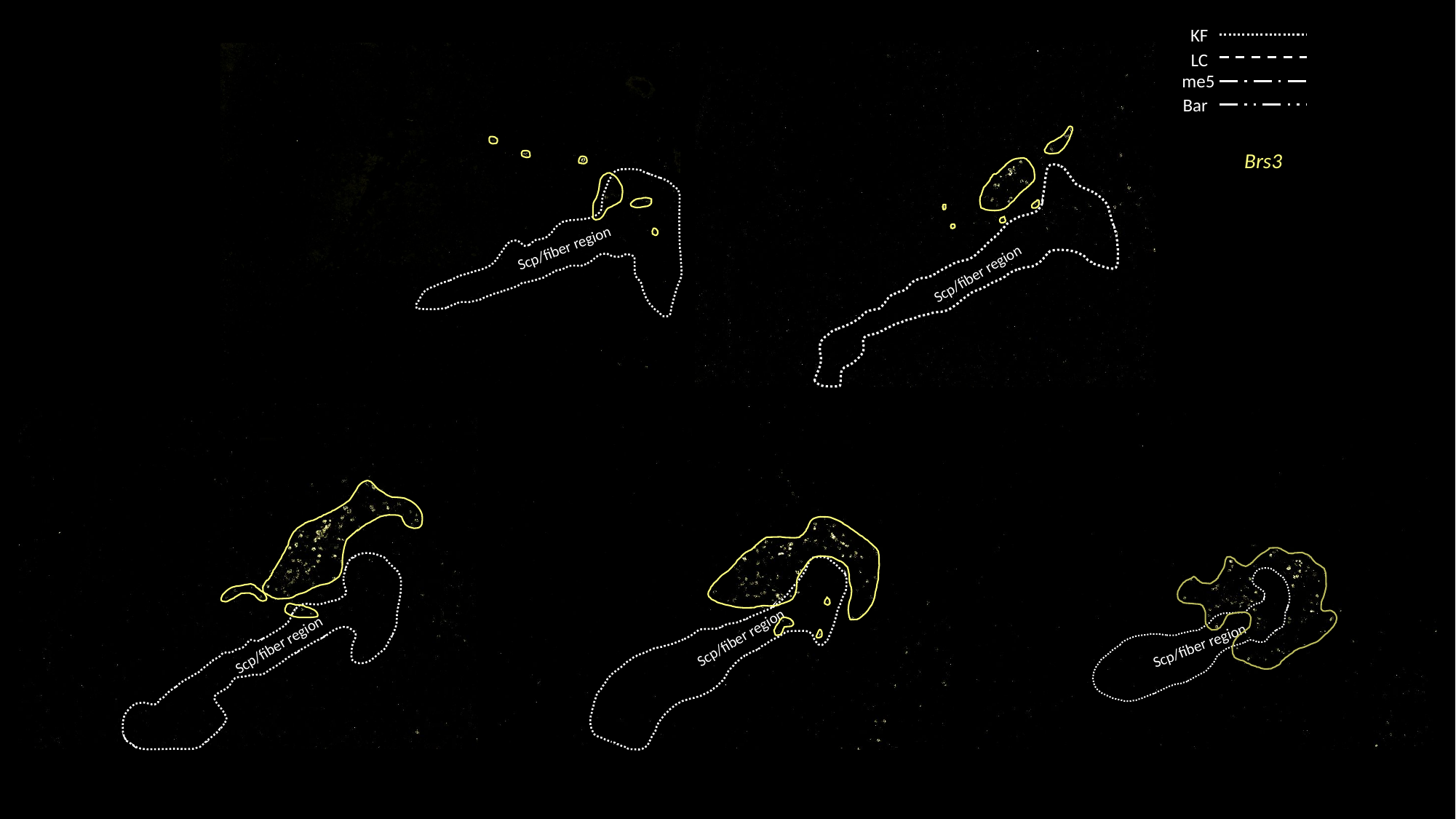

KF
Scp/fiber region
Scp/fiber region
LC
me5
Bar
Brs3
Scp/fiber region
Scp/fiber region
Scp/fiber region

## Slide 4
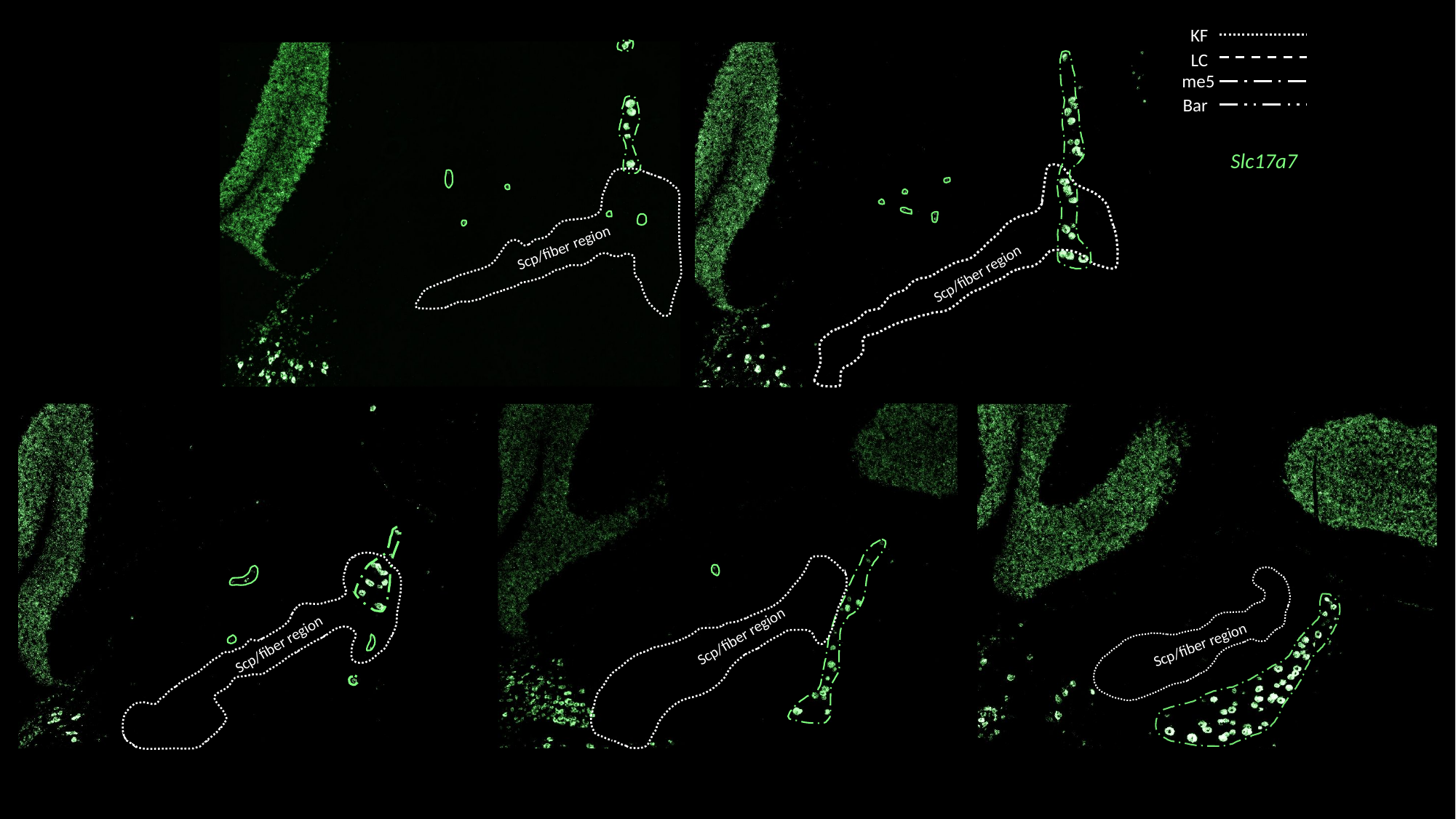

KF
Scp/fiber region
Scp/fiber region
LC
me5
Bar
Slc17a7
Scp/fiber region
Scp/fiber region
Scp/fiber region

## Slide 5
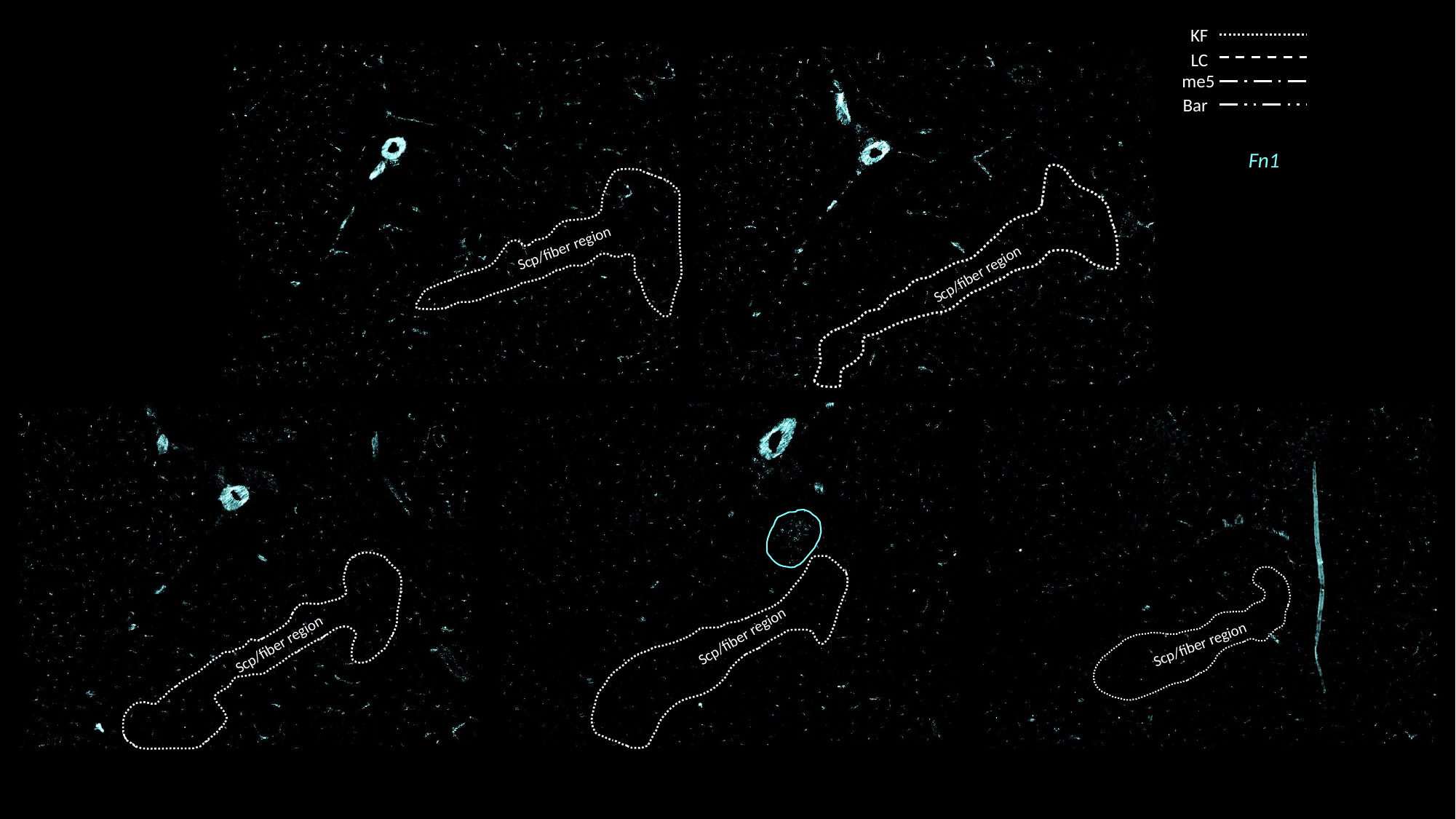

KF
Scp/fiber region
Scp/fiber region
LC
me5
Bar
Fn1
Scp/fiber region
Scp/fiber region
Scp/fiber region

## Slide 6
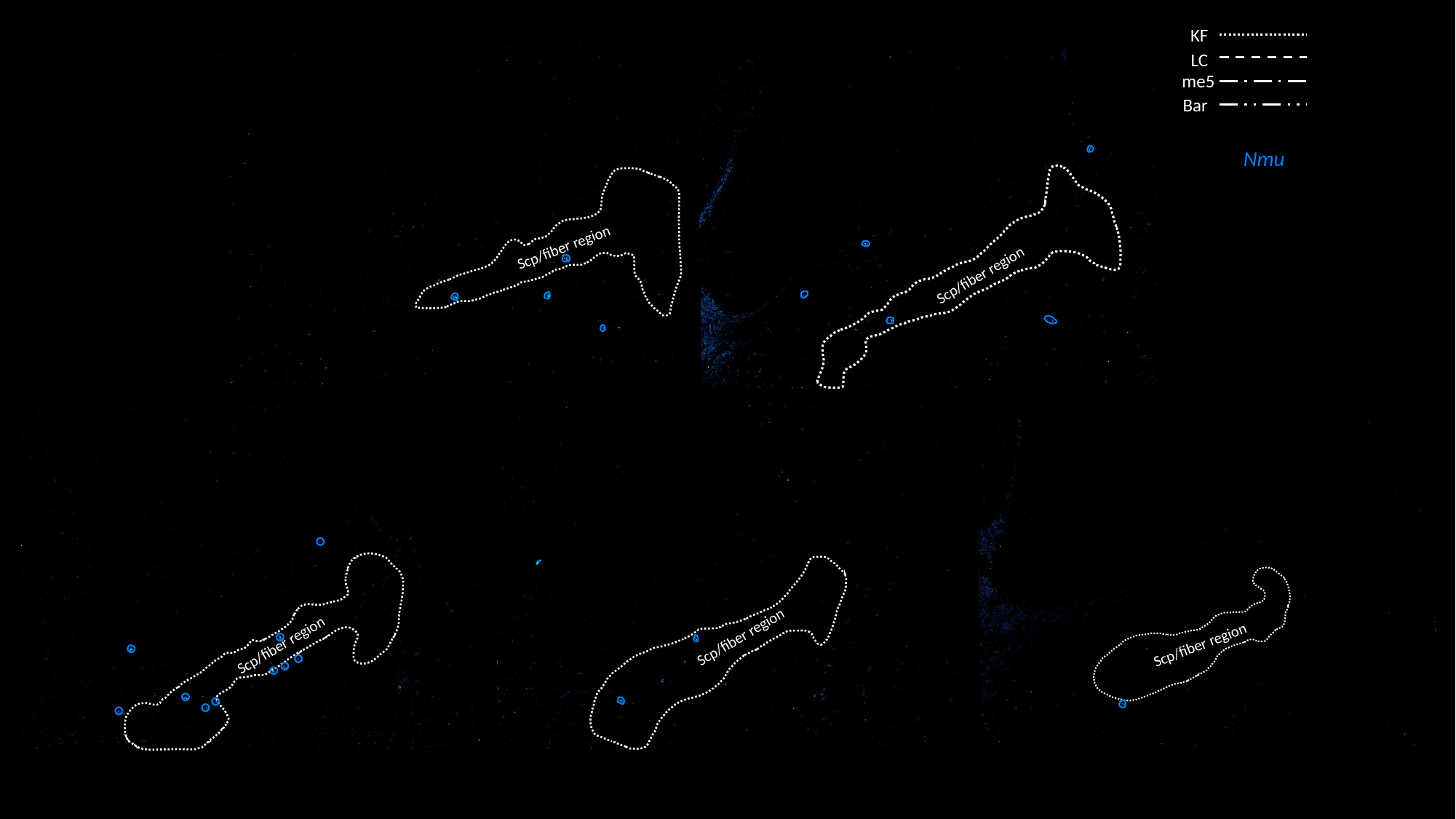

KF
LC
me5
Bar
Nmu
Scp/fiber region
Scp/fiber region
Scp/fiber region
Scp/fiber region
Scp/fiber region

## Slide 7
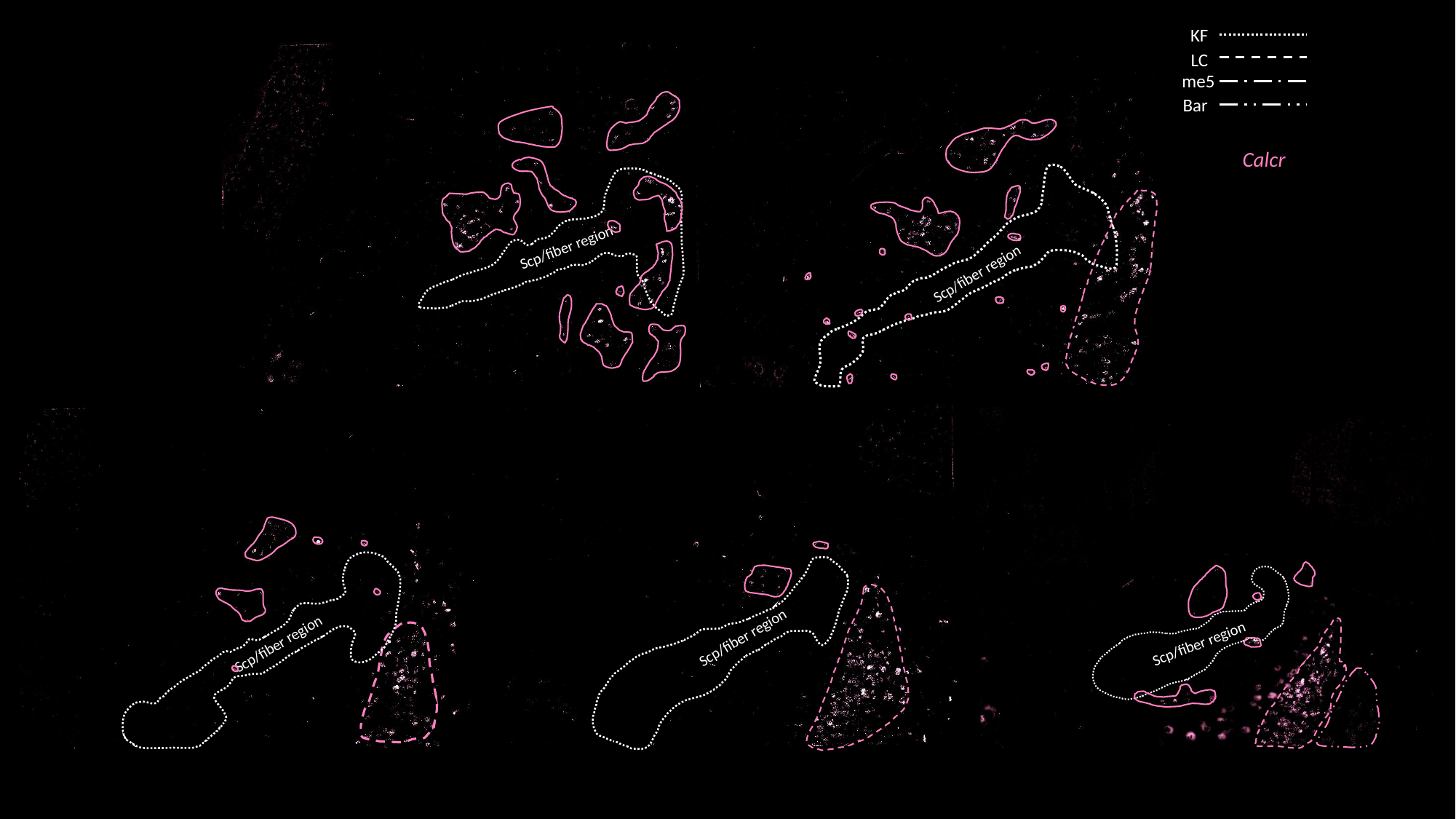

KF
LC
me5
Bar
Scp/fiber region
Calcr
Scp/fiber region
Scp/fiber region
Scp/fiber region
Scp/fiber region

## Slide 8
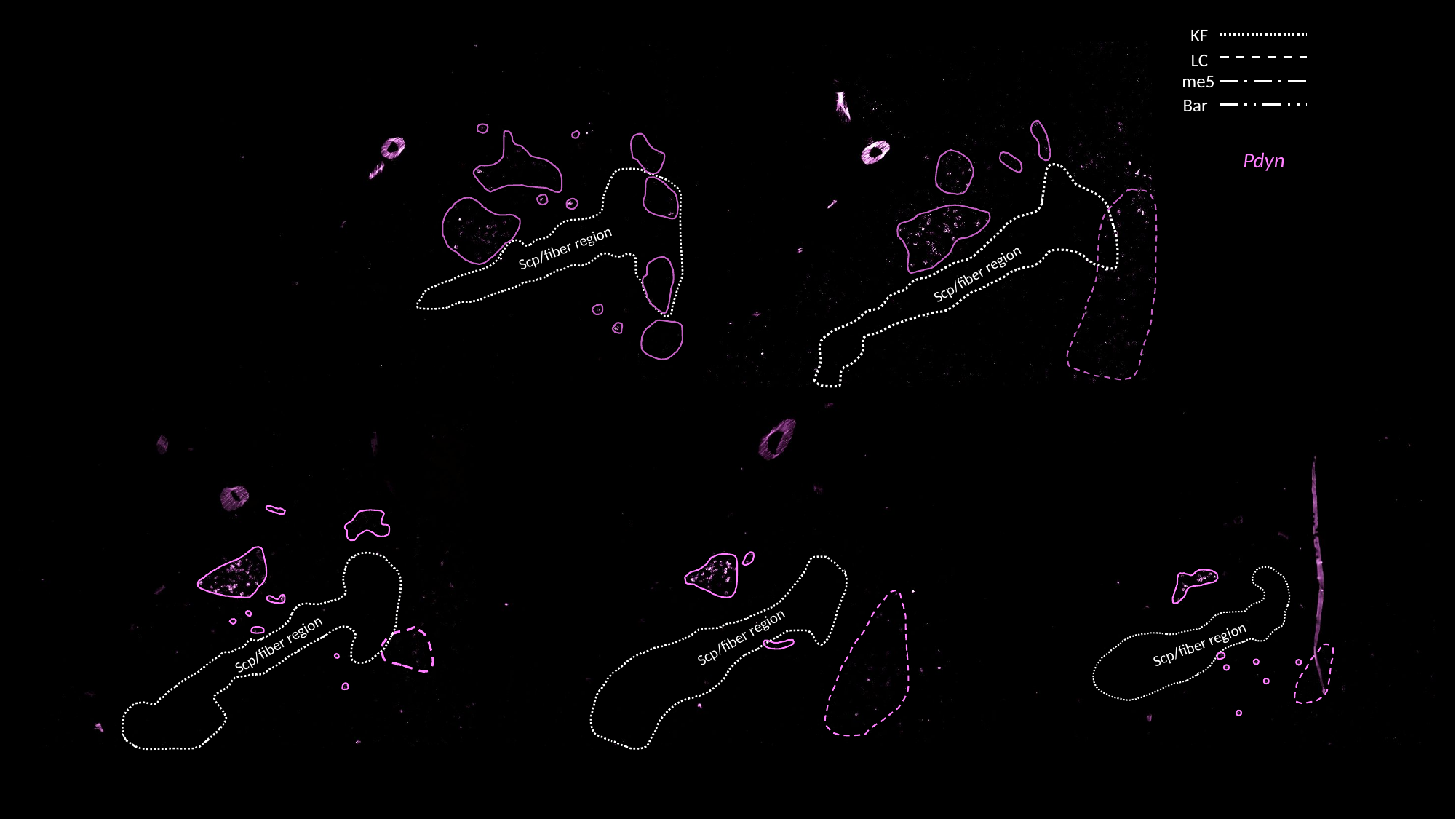

KF
Scp/fiber region
Scp/fiber region
LC
me5
Bar
Pdyn
Scp/fiber region
Scp/fiber region
Scp/fiber region

## Slide 9
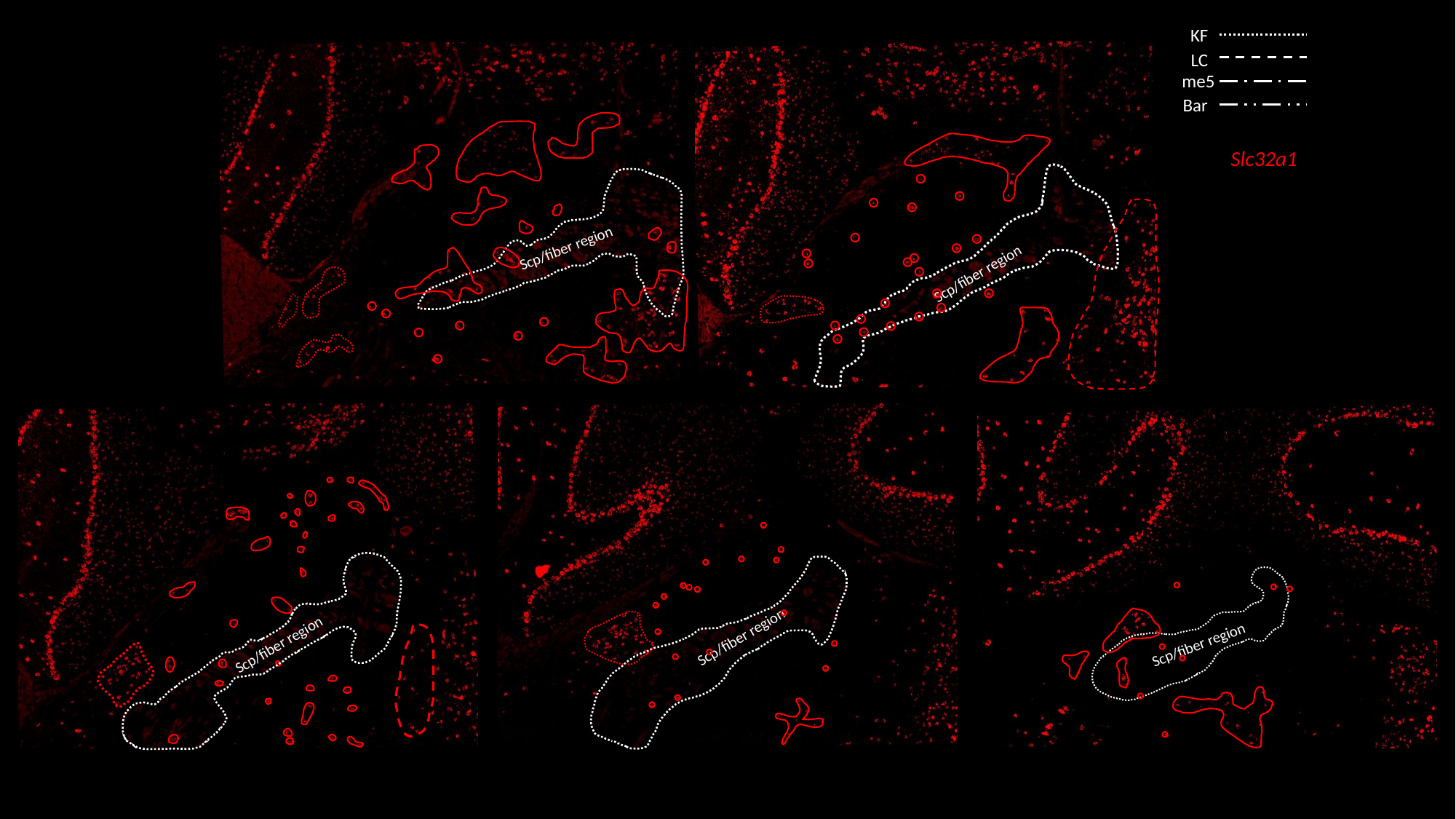

KF
Scp/fiber region
Scp/fiber region
LC
me5
Bar
Slc32a1
Scp/fiber region
Scp/fiber region
Scp/fiber region

## Slide 10
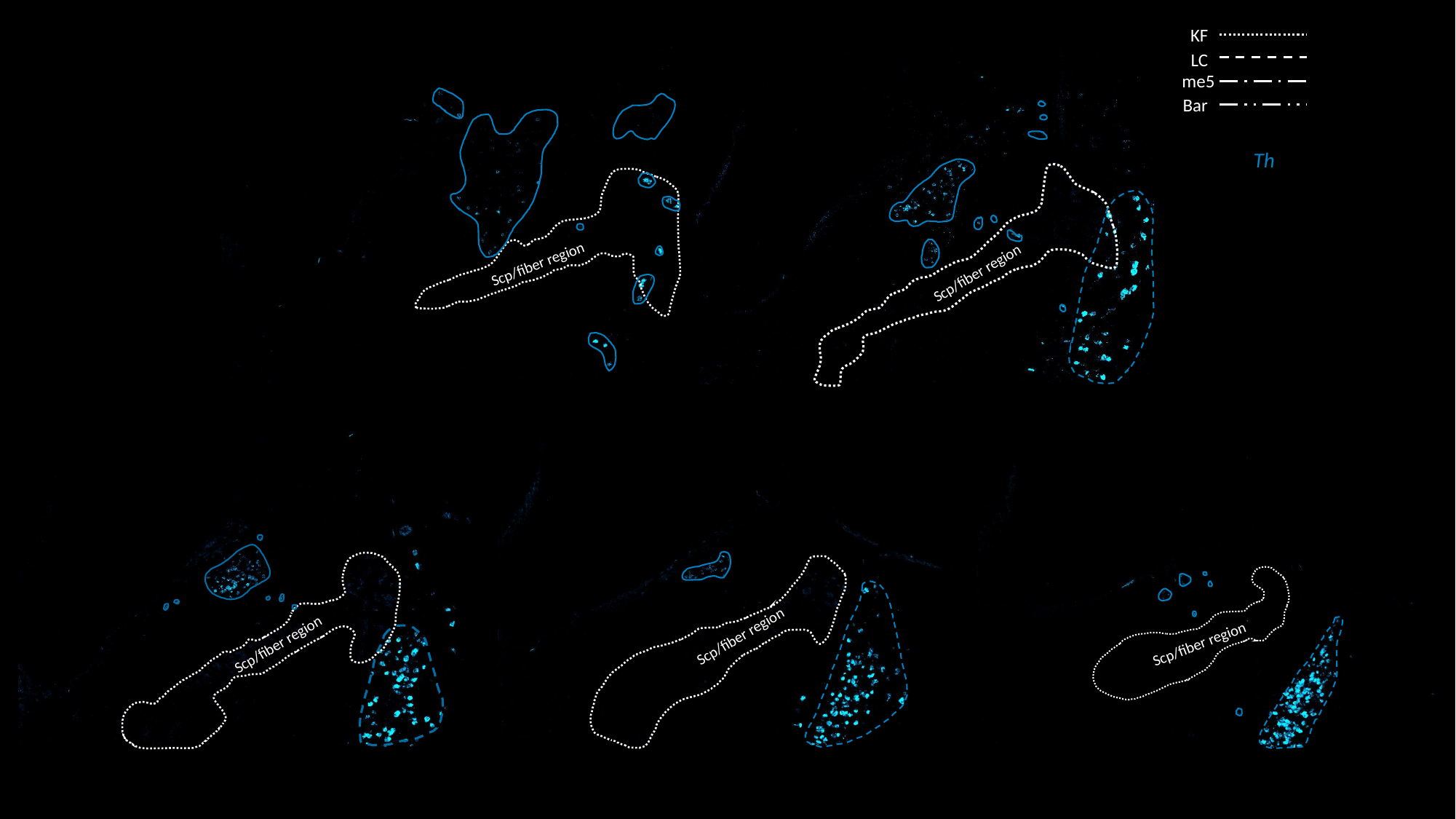

KF
Scp/fiber region
LC
me5
Bar
Scp/fiber region
Th
Scp/fiber region
Scp/fiber region
Scp/fiber region

## Slide 11
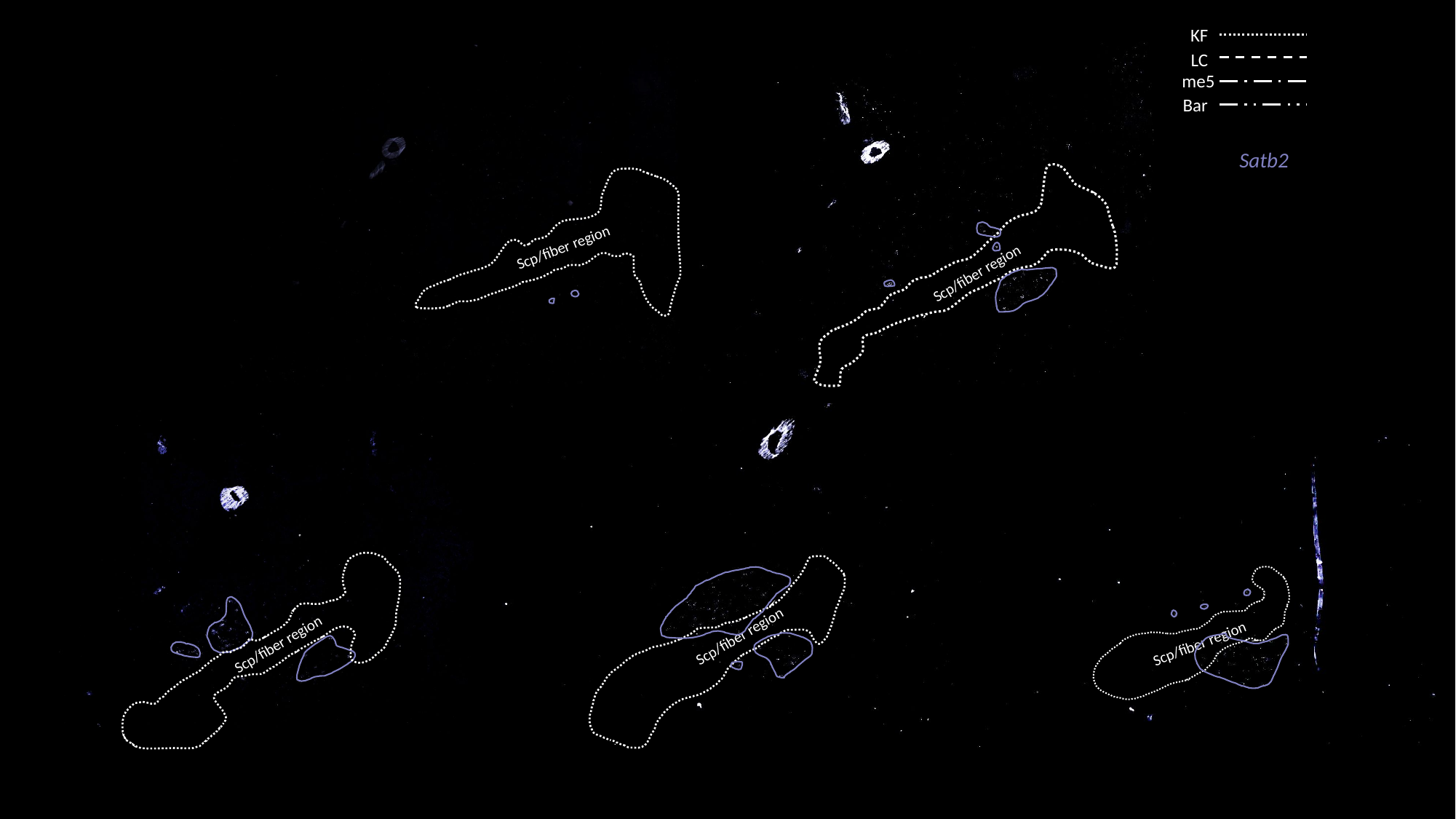

KF
Scp/fiber region
Scp/fiber region
LC
me5
Bar
Satb2
Scp/fiber region
Scp/fiber region
Scp/fiber region

## Slide 12
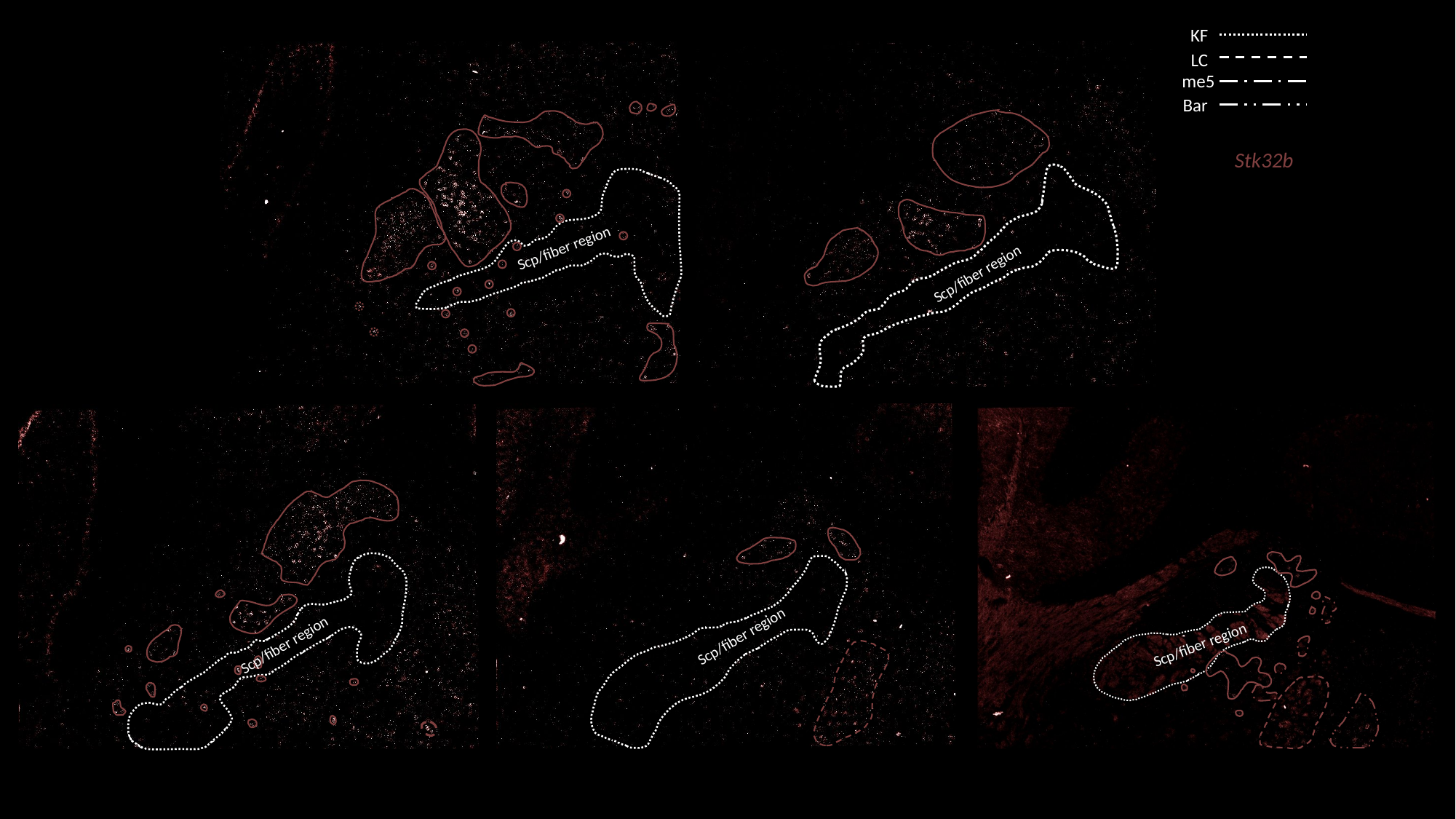

KF
LC
me5
Bar
Scp/fiber region
Stk32b
Scp/fiber region
Scp/fiber region
Scp/fiber region
Scp/fiber region

## Slide 13
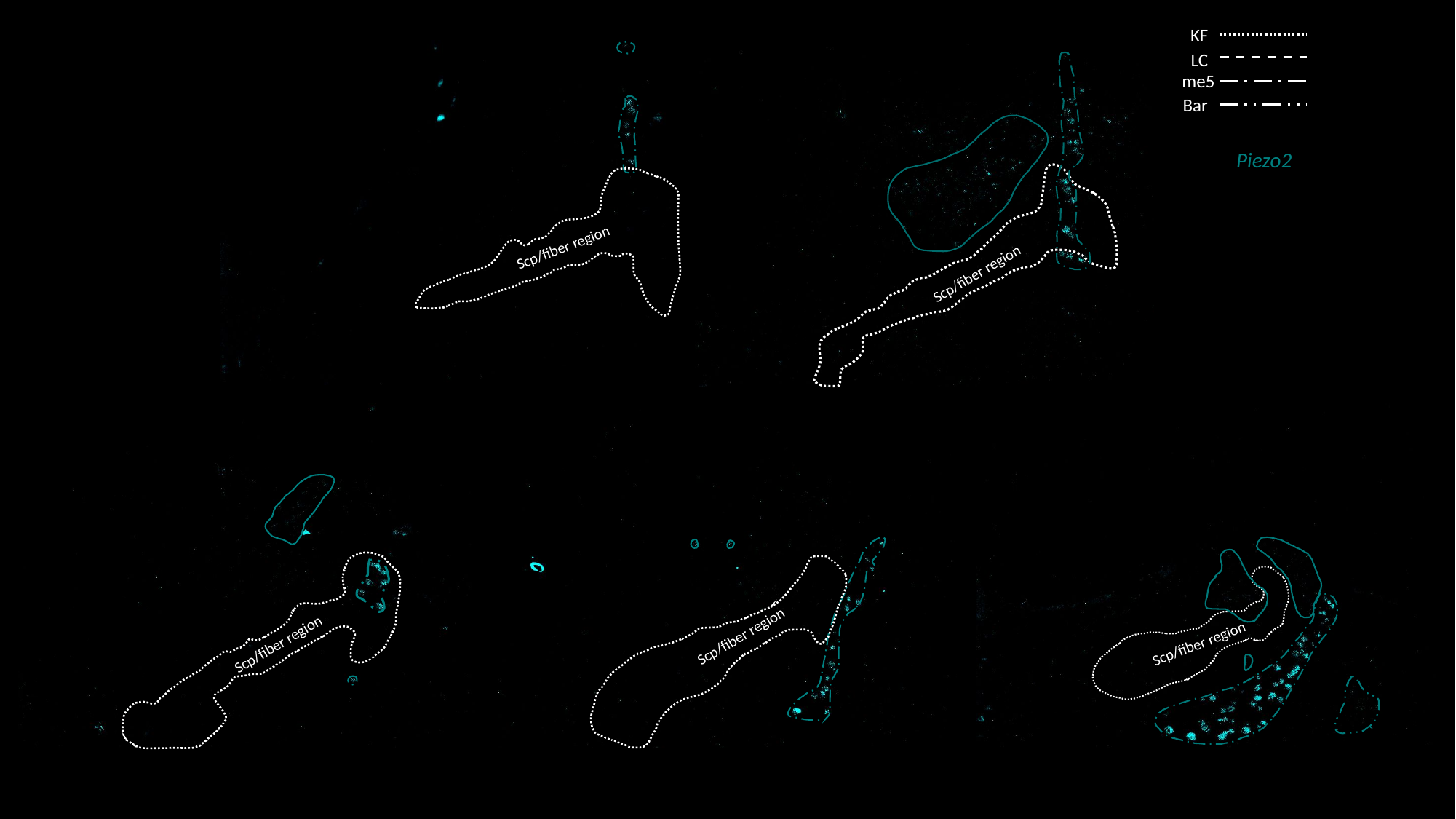

KF
Scp/fiber region
Scp/fiber region
LC
me5
Bar
Piezo2
Scp/fiber region
Scp/fiber region
Scp/fiber region

## Slide 14
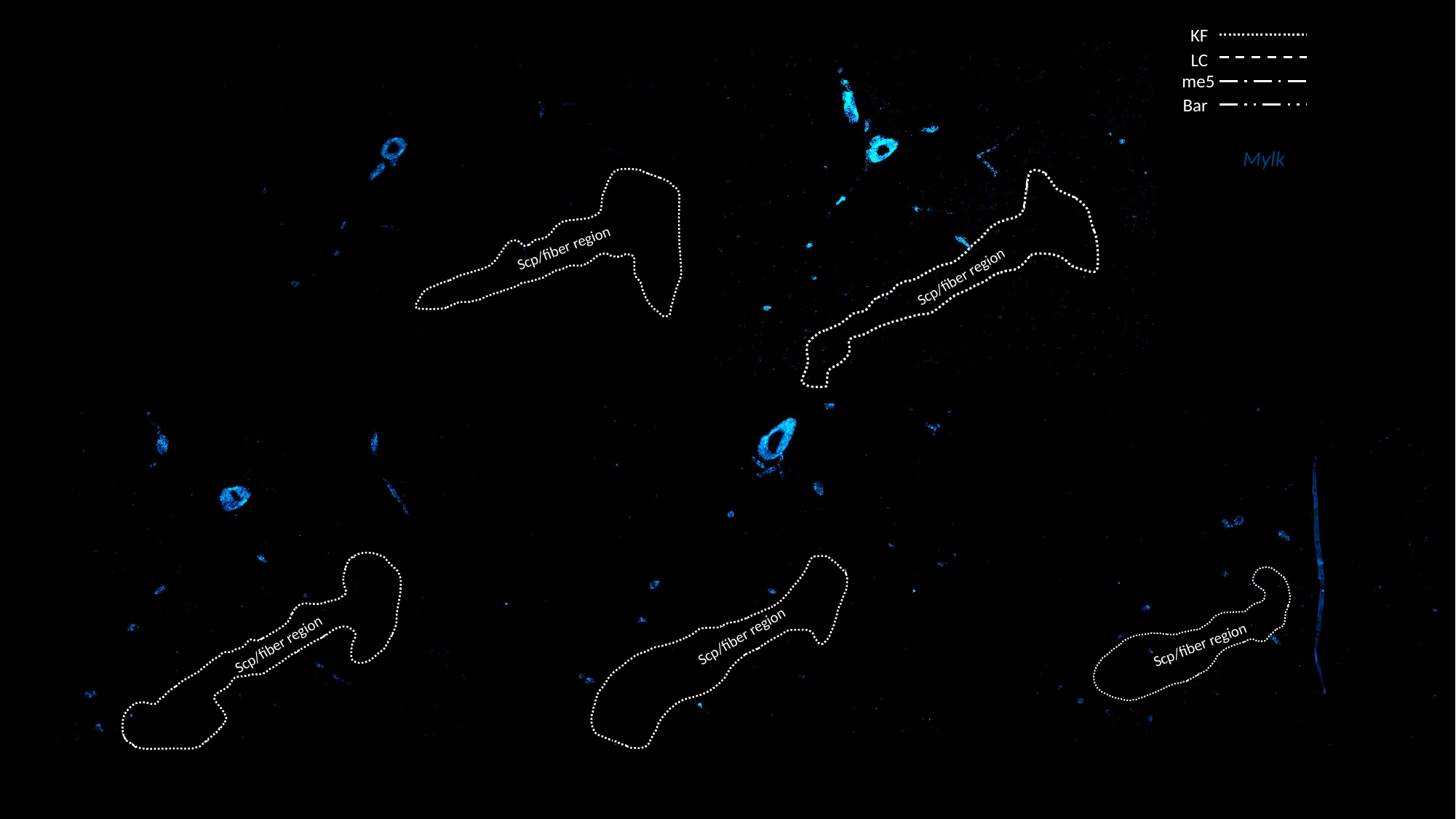

KF
Scp/fiber region
Scp/fiber region
LC
me5
Bar
Mylk
Scp/fiber region
Scp/fiber region
Scp/fiber region

## Slide 15
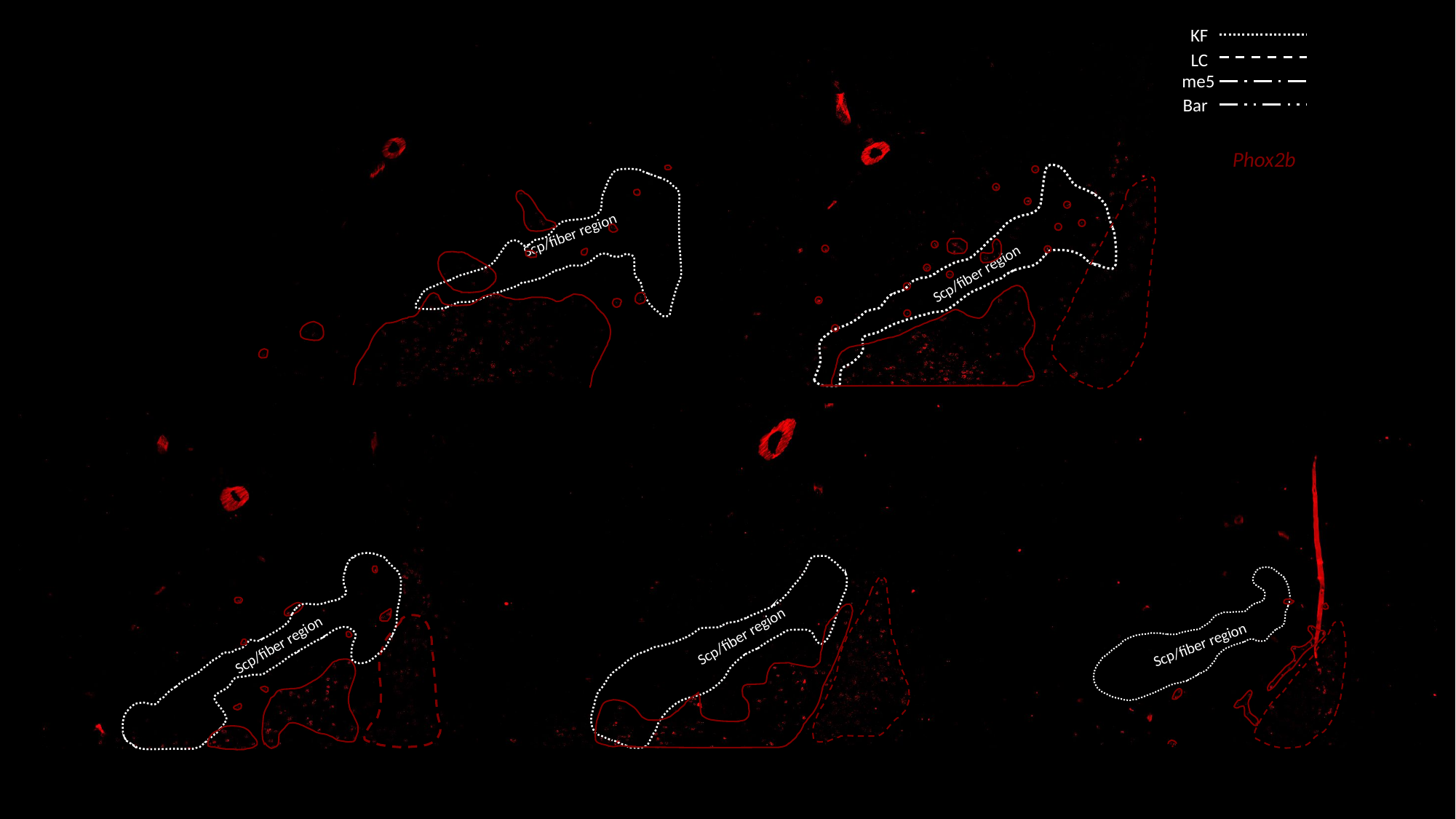

KF
Scp/fiber region
Scp/fiber region
LC
me5
Bar
Phox2b
Scp/fiber region
Scp/fiber region
Scp/fiber region

## Slide 16
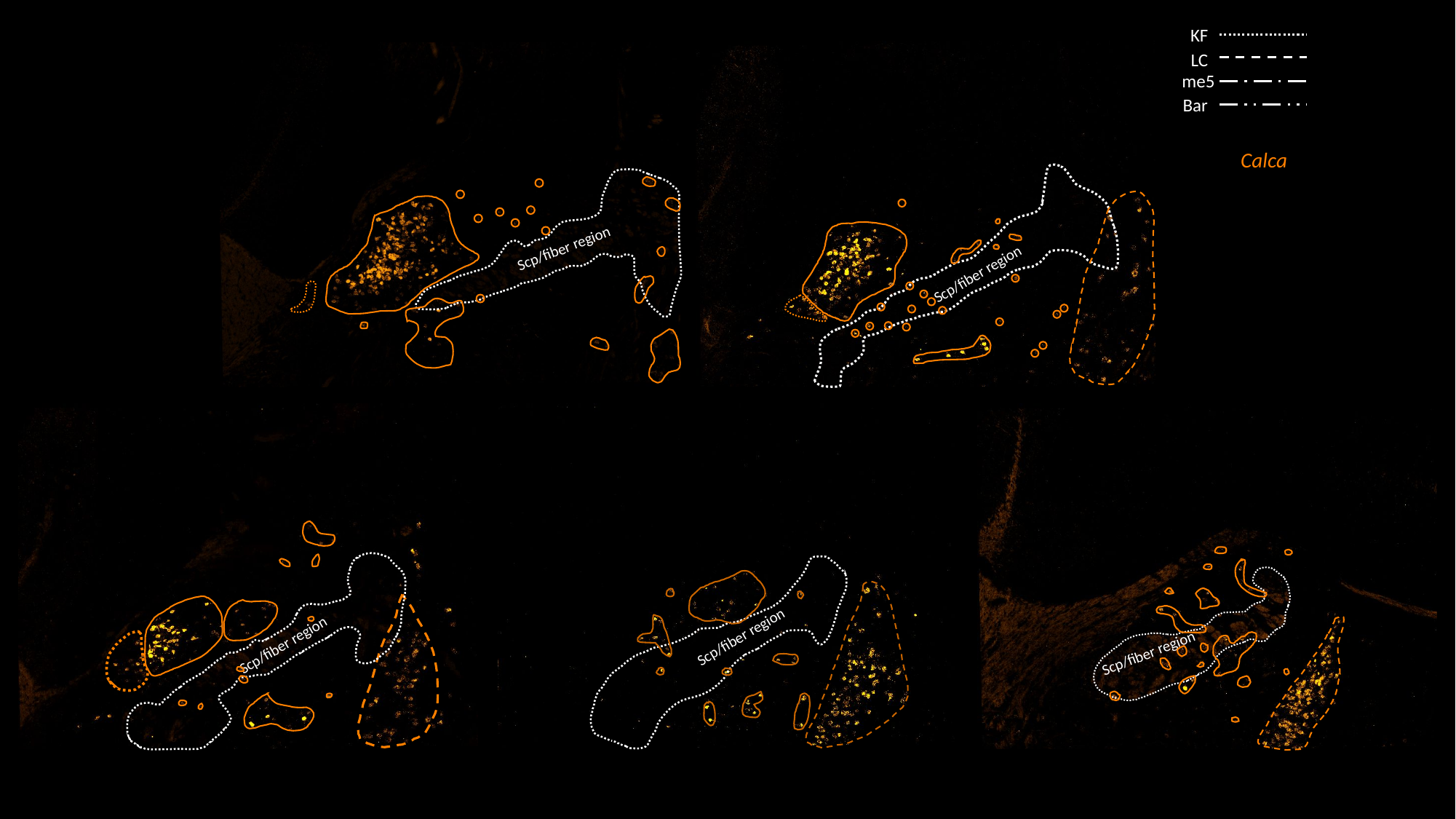

KF
LC
me5
Bar
Calca
Scp/fiber region
Scp/fiber region
Scp/fiber region
Scp/fiber region
Scp/fiber region

## Slide 17
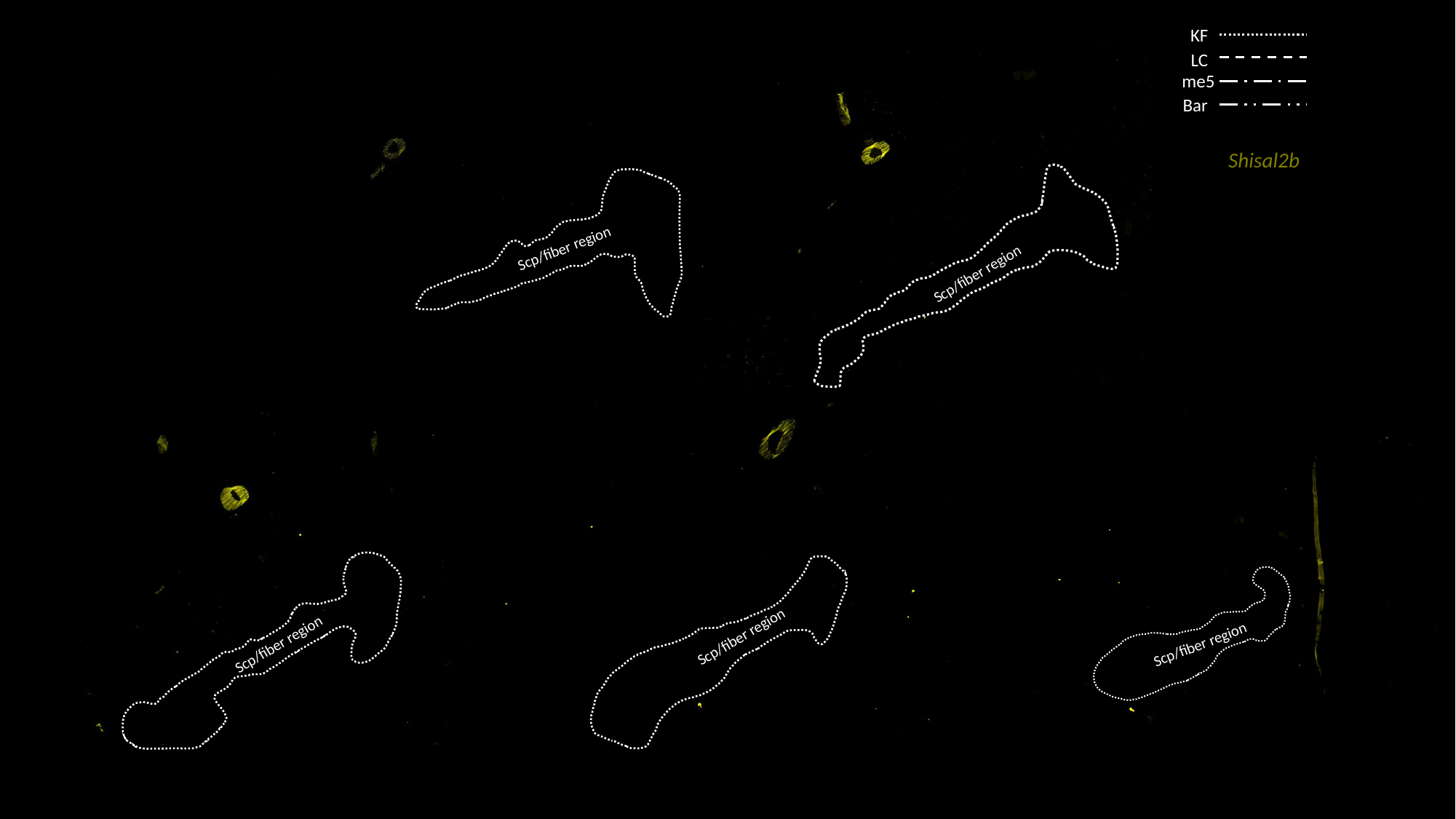

KF
Scp/fiber region
Scp/fiber region
LC
me5
Bar
Shisal2b
Scp/fiber region
Scp/fiber region
Scp/fiber region

## Slide 18
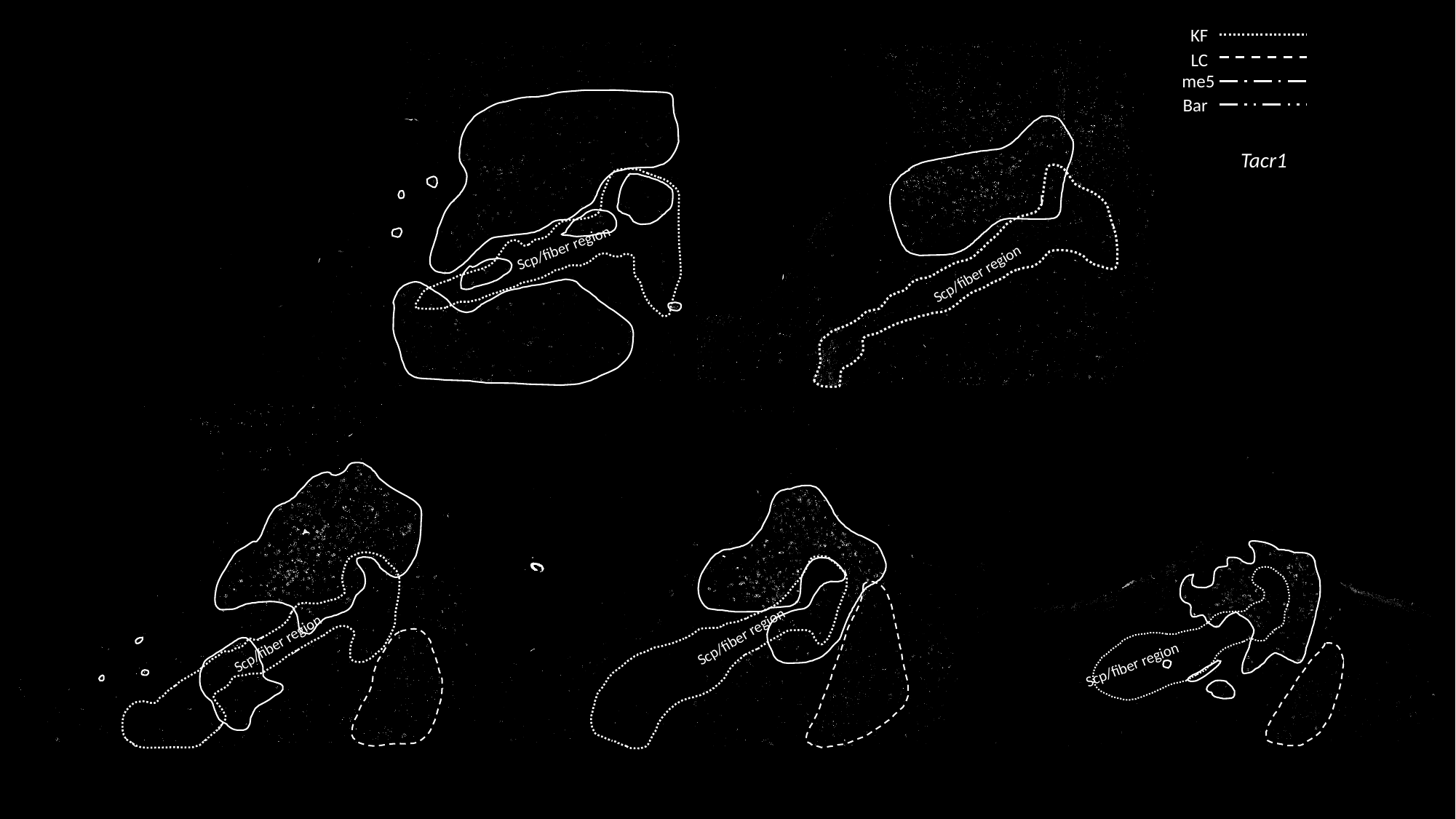

KF
Scp/fiber region
LC
me5
Bar
Scp/fiber region
Tacr1
Scp/fiber region
Scp/fiber region
Scp/fiber region

## Slide 19
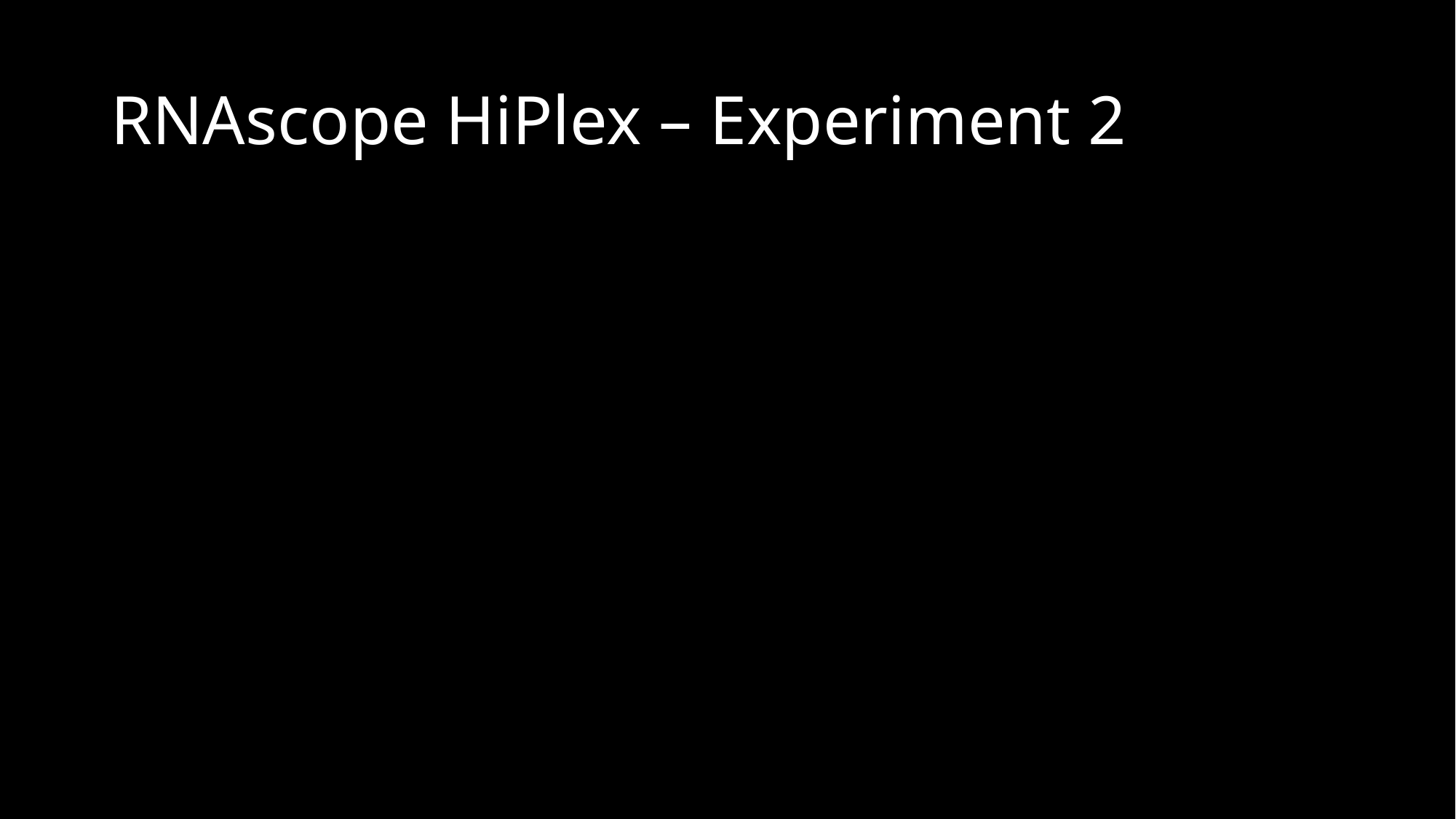

# RNAscope HiPlex – Experiment 2

## Slide 20
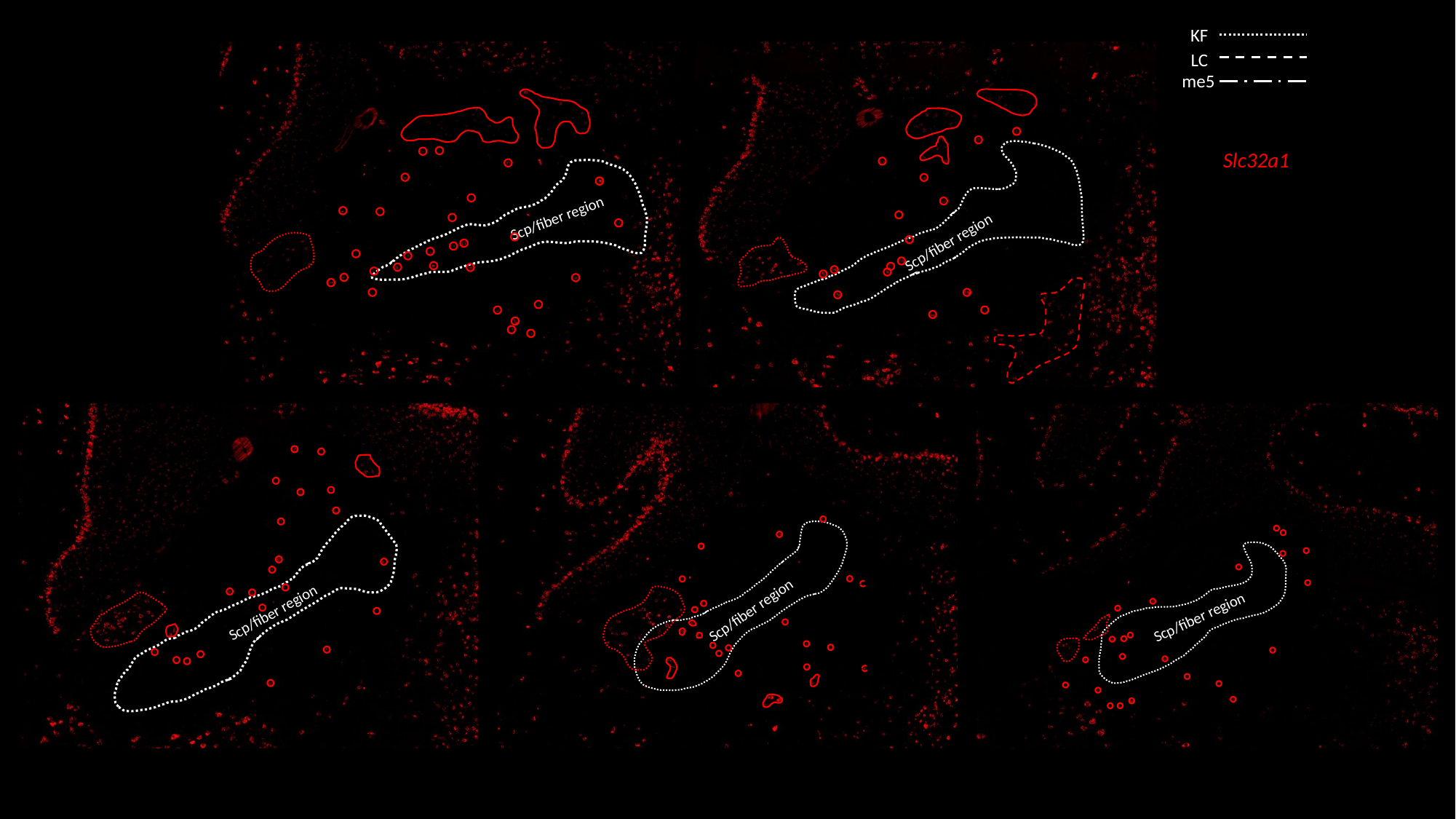

KF
Scp/fiber region
Scp/fiber region
LC
me5
Slc32a1
Scp/fiber region
Scp/fiber region
Scp/fiber region

## Slide 21
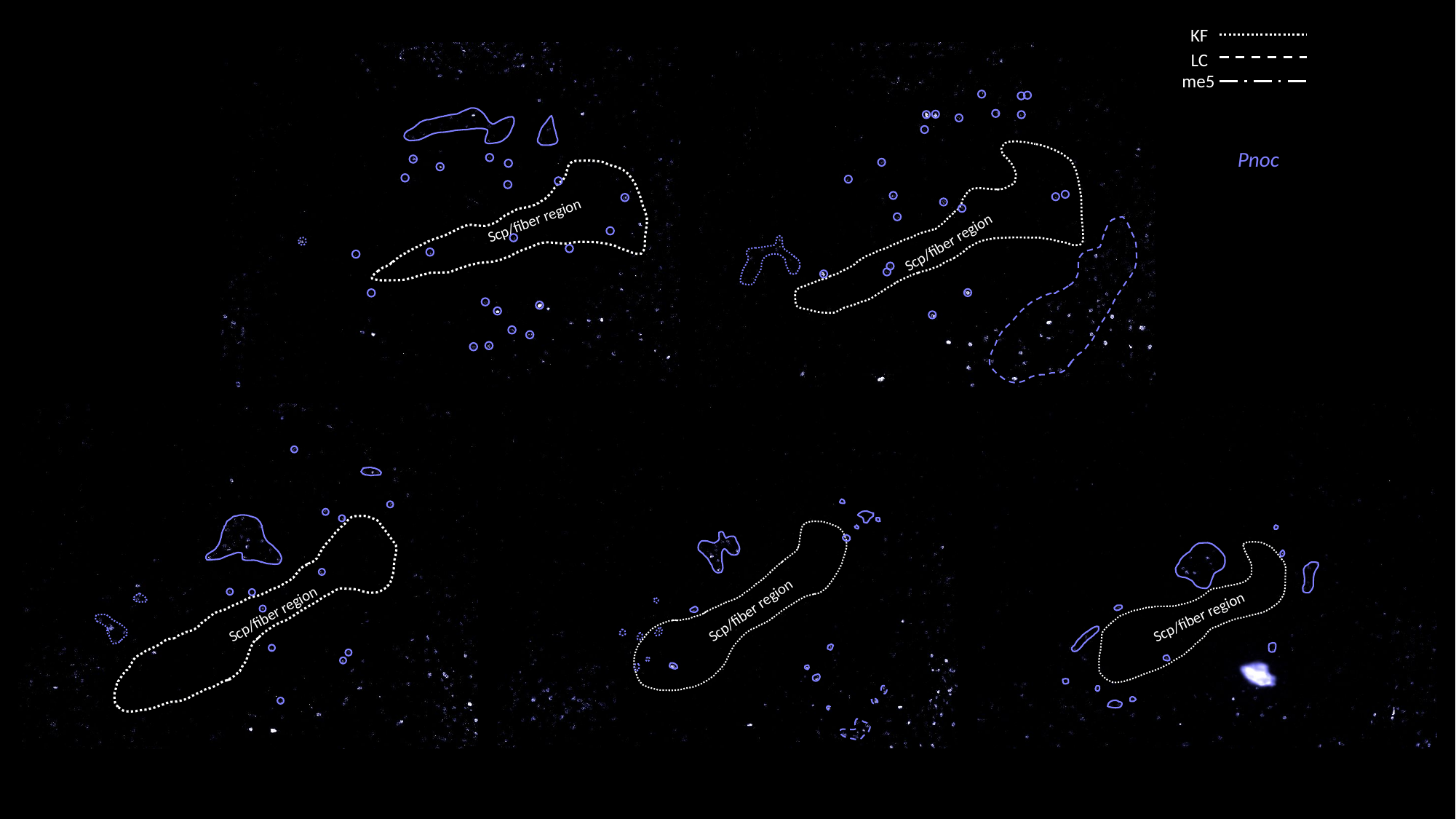

KF
Scp/fiber region
Scp/fiber region
LC
me5
Pnoc
Scp/fiber region
Scp/fiber region
Scp/fiber region

## Slide 22
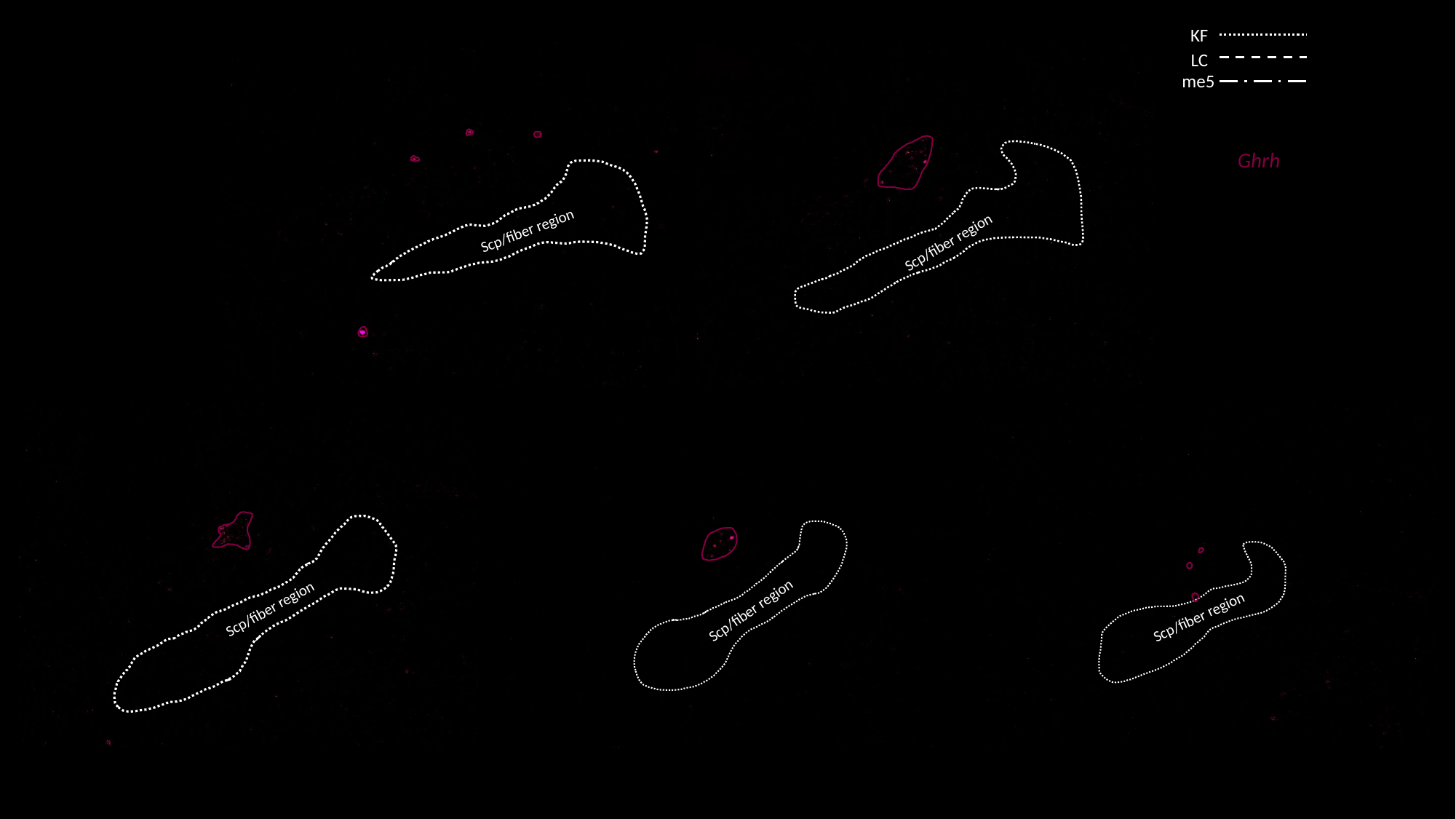

KF
Scp/fiber region
Scp/fiber region
LC
me5
Ghrh
Scp/fiber region
Scp/fiber region
Scp/fiber region

## Slide 23
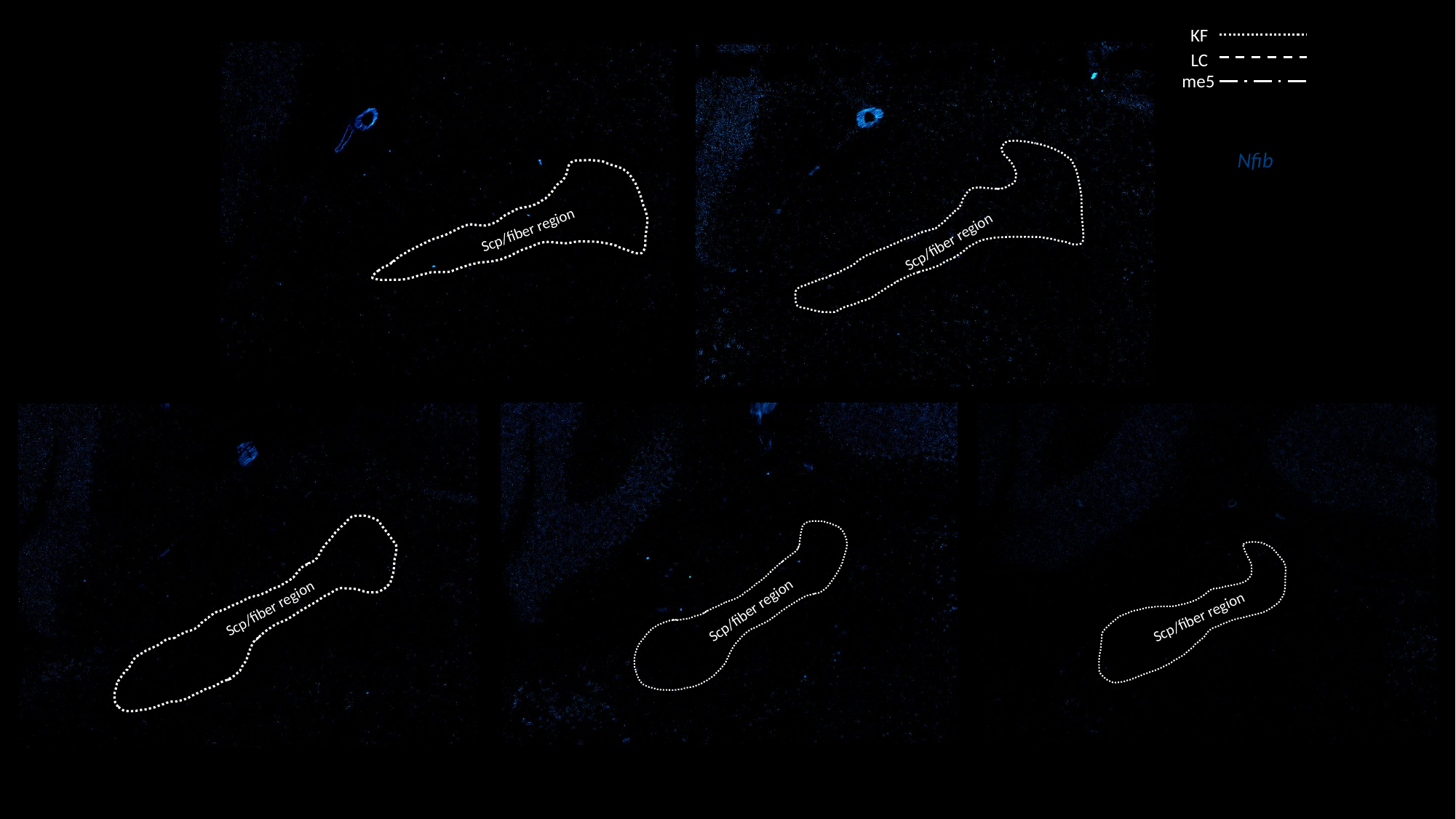

KF
Scp/fiber region
Scp/fiber region
LC
me5
Nfib
Scp/fiber region
Scp/fiber region
Scp/fiber region

## Slide 24
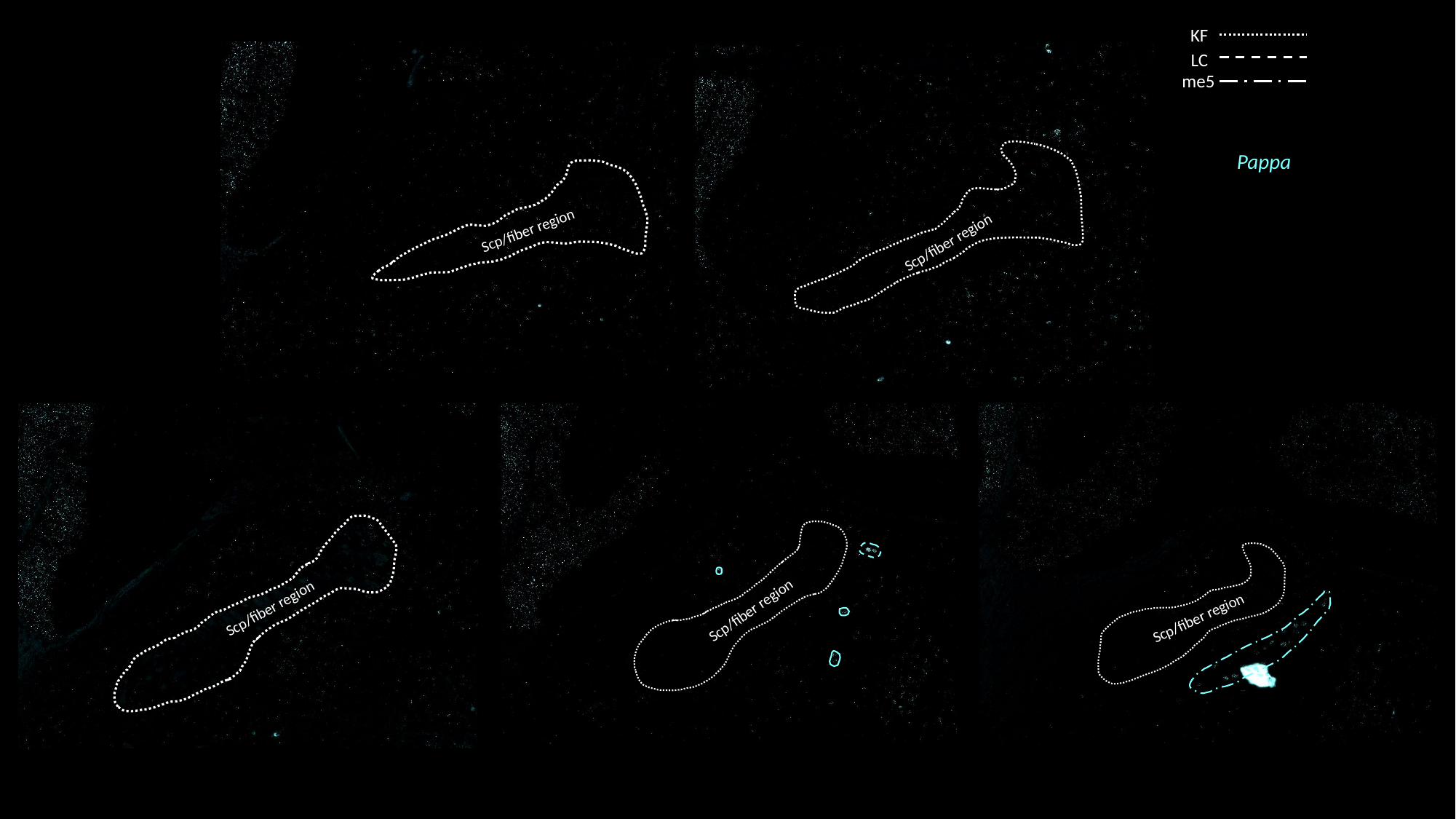

KF
Scp/fiber region
Scp/fiber region
LC
me5
Pappa
Scp/fiber region
Scp/fiber region
Scp/fiber region

## Slide 25
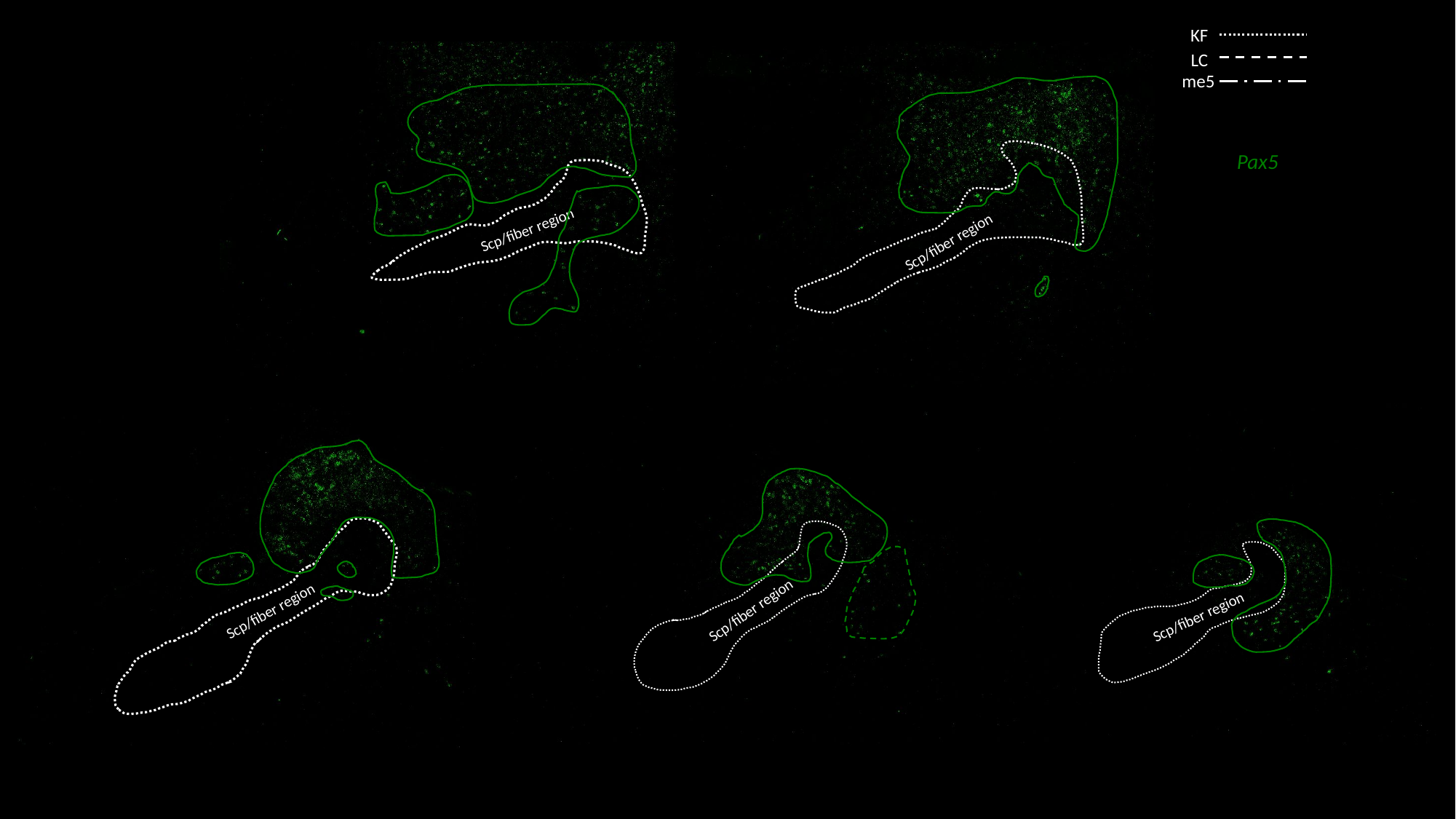

KF
Scp/fiber region
Scp/fiber region
LC
me5
Pax5
Scp/fiber region
Scp/fiber region
Scp/fiber region

## Slide 26
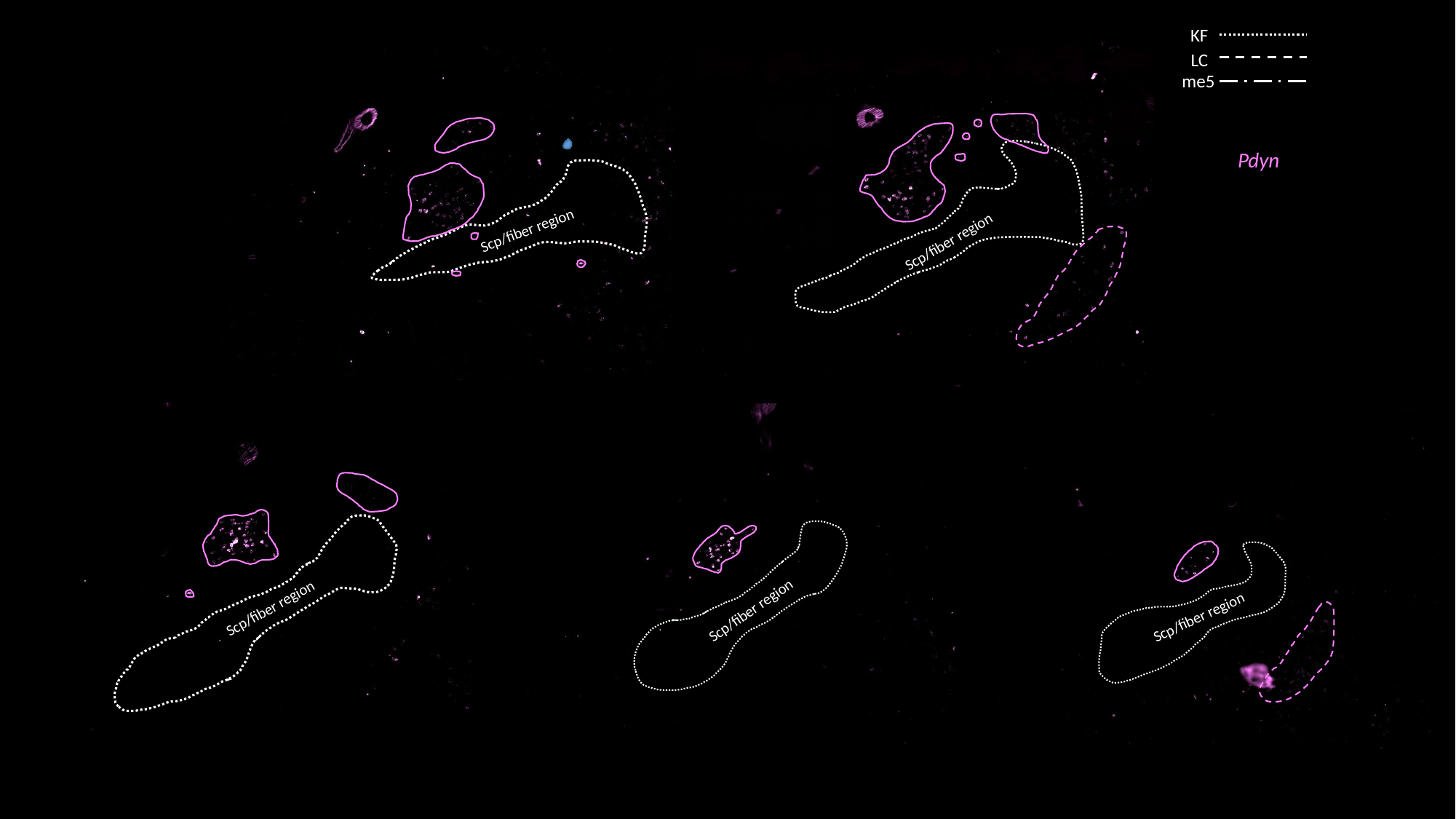

KF
Scp/fiber region
Scp/fiber region
LC
me5
Pdyn
Scp/fiber region
Scp/fiber region
Scp/fiber region

## Slide 27
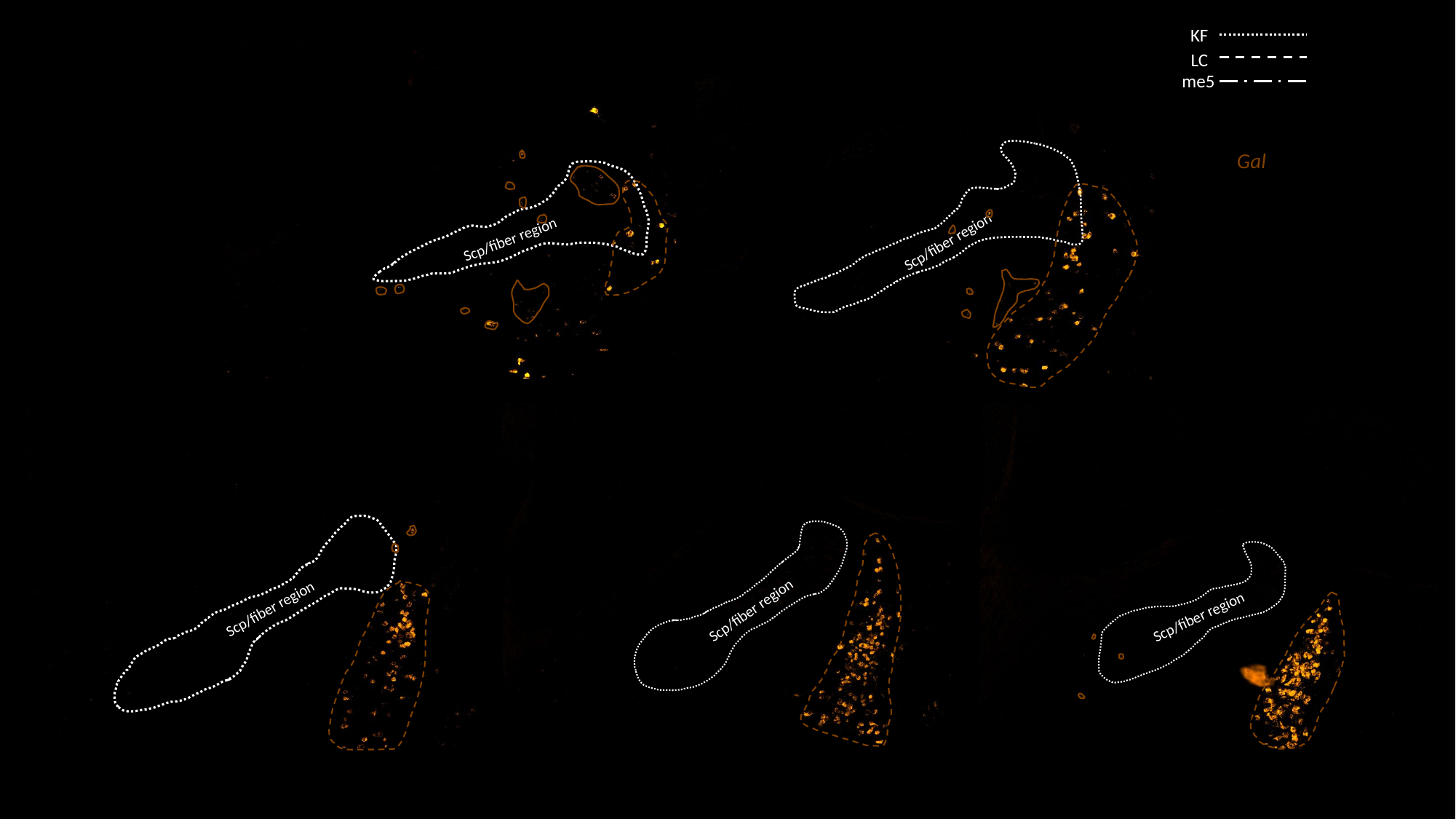

KF
Scp/fiber region
Scp/fiber region
LC
me5
Gal
Scp/fiber region
Scp/fiber region
Scp/fiber region

## Slide 28
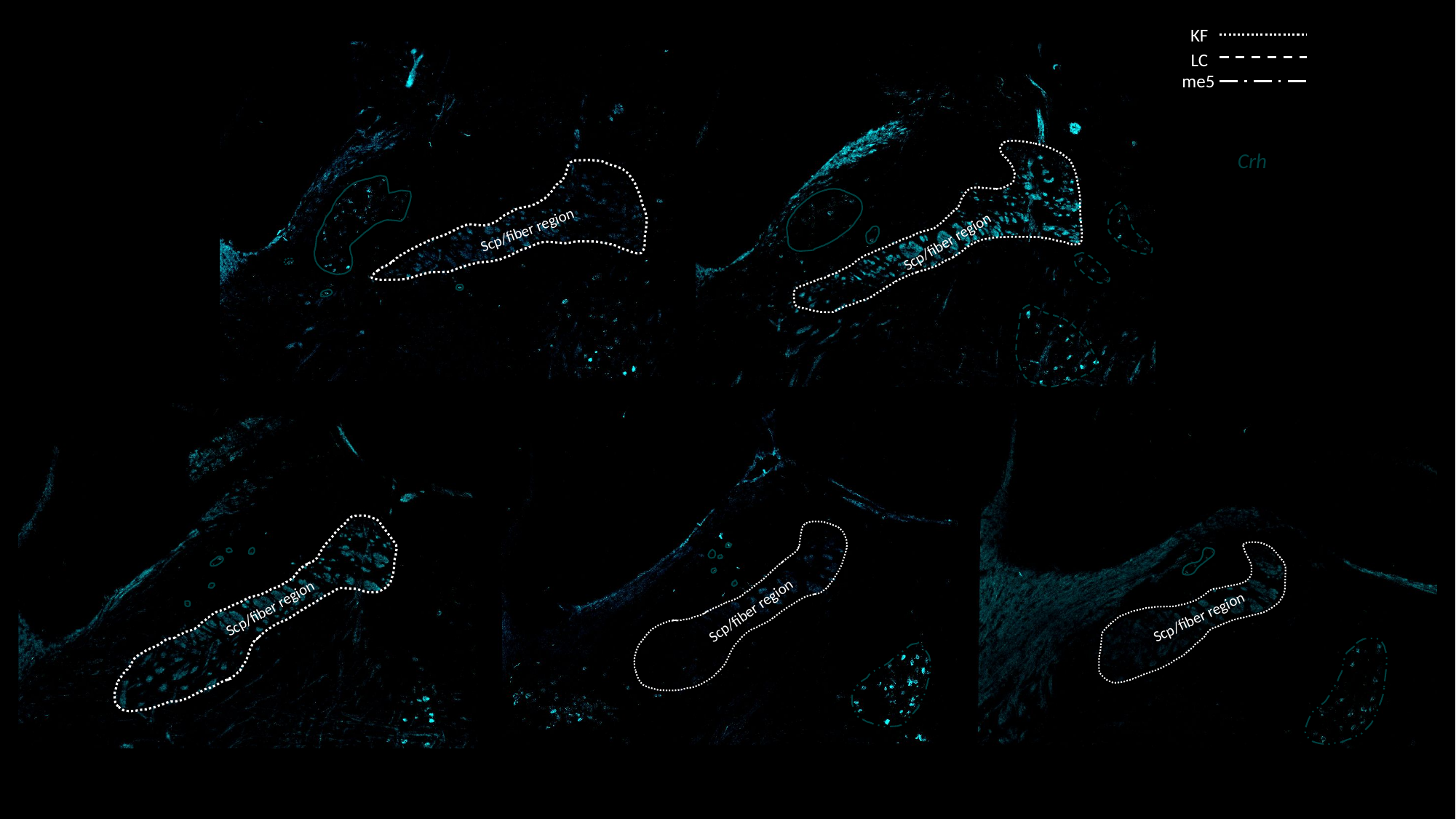

KF
Scp/fiber region
Scp/fiber region
LC
me5
Crh
Scp/fiber region
Scp/fiber region
Scp/fiber region

## Slide 29
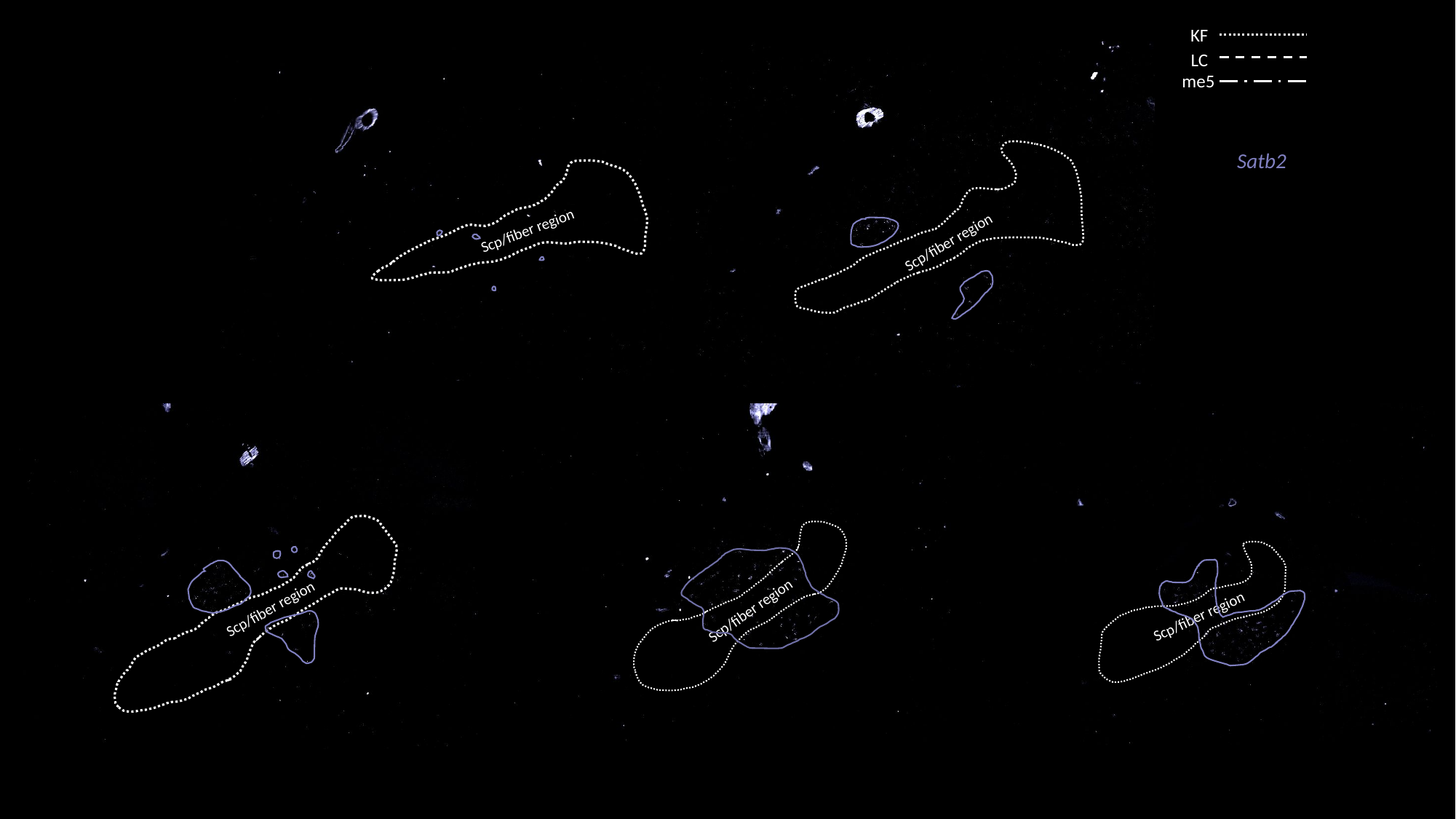

KF
Scp/fiber region
Scp/fiber region
LC
me5
Satb2
Scp/fiber region
Scp/fiber region
Scp/fiber region

## Slide 30
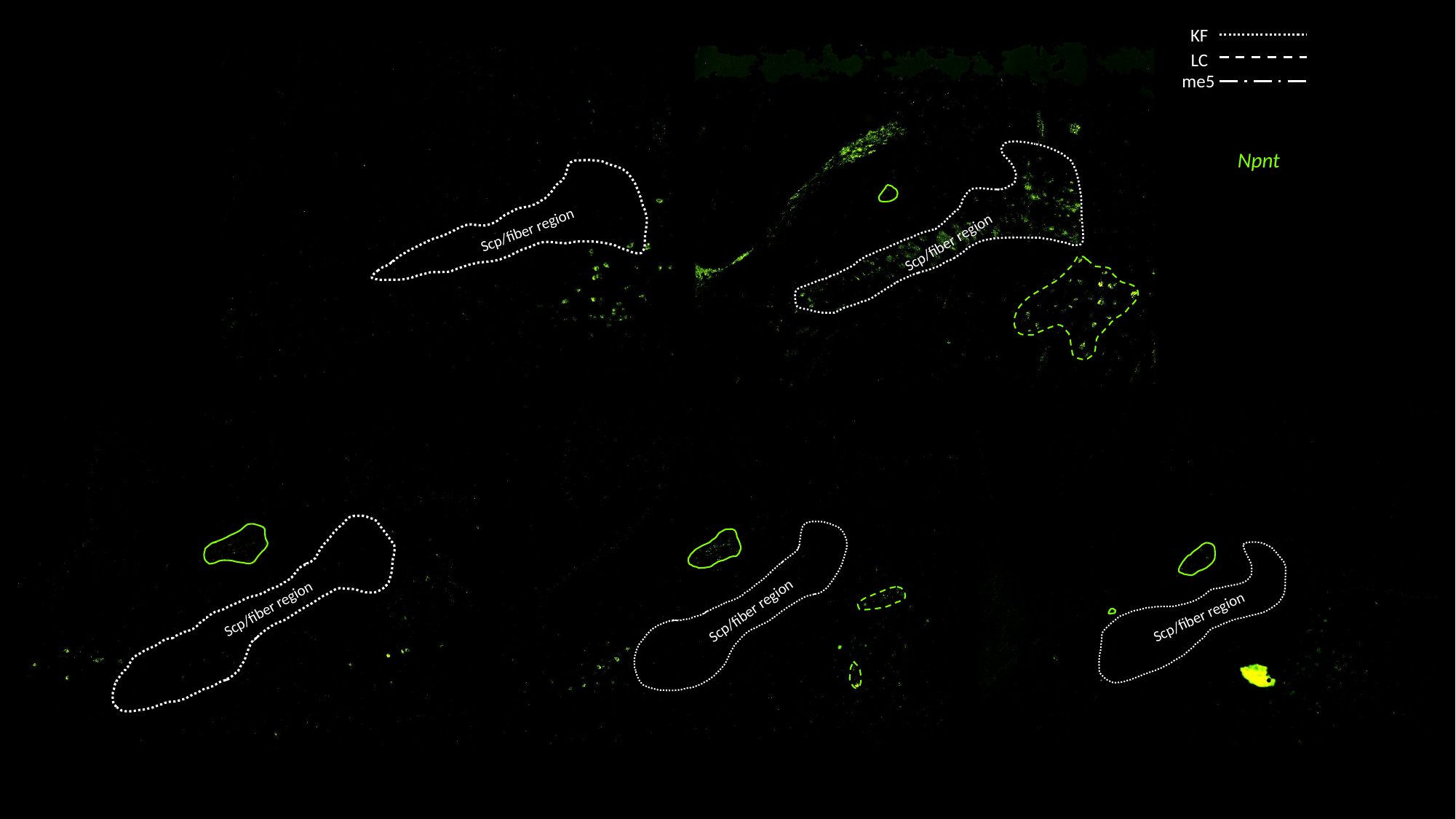

KF
Scp/fiber region
Scp/fiber region
LC
me5
Npnt
Scp/fiber region
Scp/fiber region
Scp/fiber region

## Slide 31
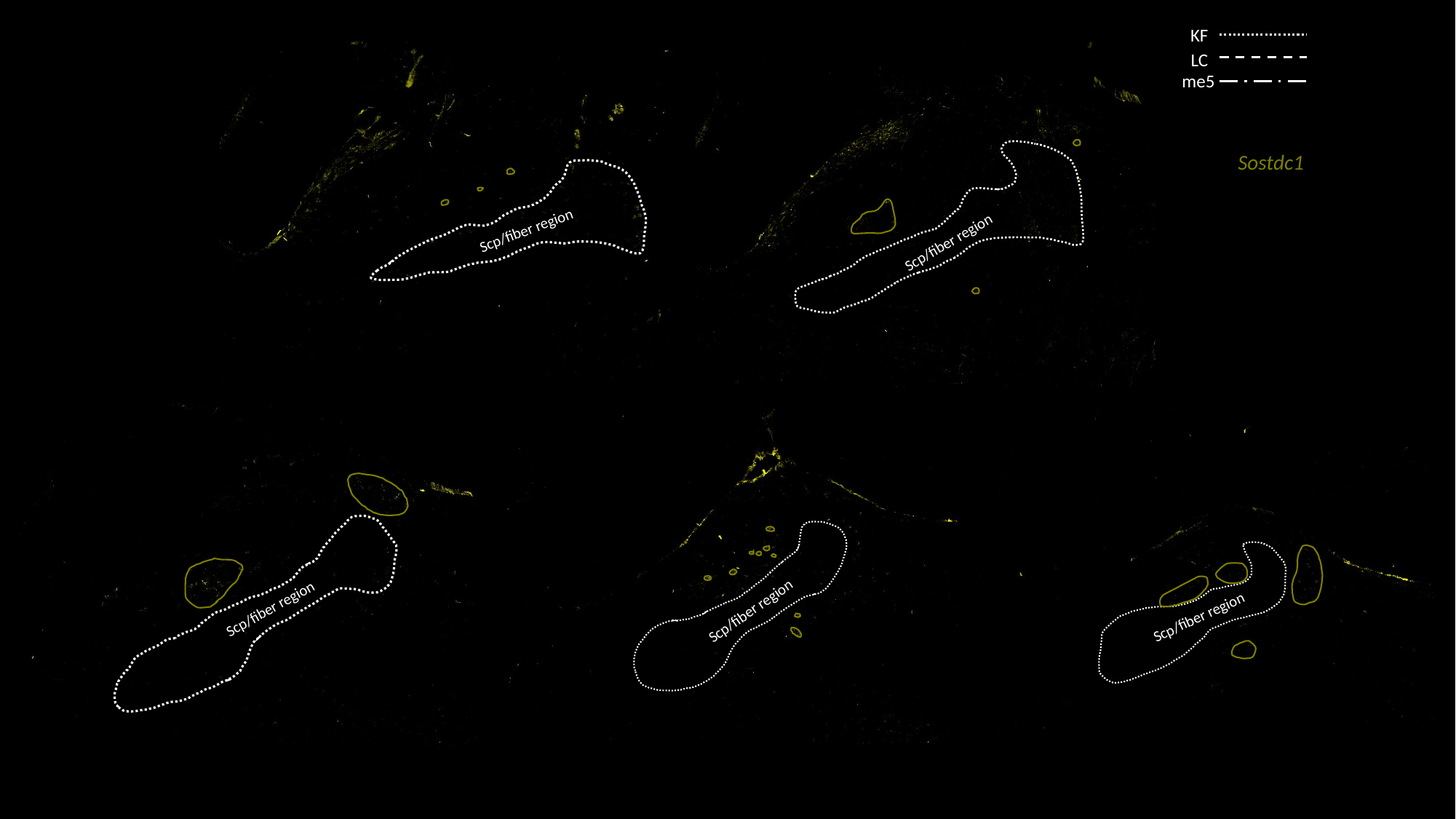

KF
Scp/fiber region
Scp/fiber region
LC
me5
Sostdc1
Scp/fiber region
Scp/fiber region
Scp/fiber region
